# Hybrid Nano‐Atomic IrFe–N–C for Low‐Iridium Oxygen Electrocatalysis

**DOI:** 10.1002/smsc.70358

**Published:** 2026-07-29

**Authors:** Meng‐Meng Liu, Han Gao, Shi‐Yu Zhu, Meng Li, Xiao Ren, Yi‐Fang Yuan, Wei‐Jie Liu, Ling‐Rui Wang, Tian‐Yu Xia, Xiao‐Lei Shi, Hai‐Zhong Guo, Zhi‐Gang Chen

**Affiliations:** ^1^ School of Physics Zhengzhou University Zhengzhou China; ^2^ School of Chemistry and Physics ARC Research Hub in Zero‐emission Power Generation for Carbon Neutrality and Centre for Materials Science Queensland University of Technology Brisbane Queensland Australia; ^3^ Institute of Quantum Materials and Physics Henan Academy of Sciences Zhengzhou China

**Keywords:** bifunctional oxygen electrocatalysis, Fe–N_4_, hierarchical active‐site catalyst, metal‐N–C, nanoparticles

## Abstract

Designing efficient bifunctional catalysts for oxygen evolution and oxygen reduction reactions (OER/ORR) is essential for advancing metal–air batteries, yet remains challenging due to the fundamentally distinct reaction pathways involved. Here, we address this challenge by developing a hybrid IrFe–N–C electrocatalyst that integrates IrFe nanoparticles with a Fe–N–C support rich in atomically dispersed Fe–N_4_ sites. This hierarchical active‐site design enables functional complementarity: IrFe nanoparticles preferentially catalyze the OER, while Fe–N_4_ moieties serve as highly active ORR sites. Effective metal–support interactions stabilize both single atoms and ultrafine nanoparticles, ensuring high activity and stability. As a result, the catalyst exhibits impressive bifunctional performance with reduced Ir loading, achieving an OER overpotential of only 227 mV at 10 mA cm^−2^. The mass activities for both OER and ORR far surpass those of state‐of‐the‐art Ir/C and Pt/C benchmarks. When implemented in Zn–air batteries, the catalyst delivers promising power density and fair cycling stability. These results establish a rational design strategy for cost‐effective, durable bifunctional catalysts toward next‐generation energy storage technologies.

## Graphical abstract

A hierarchical IrFe–N–C catalyst is prepared by integrating IrFe nanoparticles into a Fe–N–C matrix. The nanoparticles serve as active components for the oxygen evolution reaction, while the Fe–N_4_ sites facilitate oxygen reduction reaction. This hybrid structure enables bifunctional oxygen electrocatalysis at low iridium loading. The study emphasizes the synergistic effects of the integrated nano‐atomic components in regulating catalytic pathways.

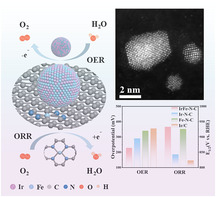

## 1 Introduction

The sluggish kinetics and large overpotentials of the oxygen reduction reaction (ORR) and oxygen evolution reaction (OER) constitute critical bottlenecks for the performance of rechargeable metal–air batteries [1, 2, 3]. However, the fundamentally distinct catalytic mechanisms and divergent electron‐transfer pathways of ORR and OER make the development of catalysts with high bifunctional activity highly desirable while intrinsically challenging [4, 5]. Ir has emerged as the benchmark electrocatalyst for OER owing to its outstanding intrinsic activity and operational stability [6, 7, 8, 9]. Nevertheless, despite their superior OER performance, Ir‐based catalysts generally exhibit inferior ORR activity compared with Pt counterparts, primarily due to suboptimal adsorption energetics and insufficient stabilization of reaction intermediates [10].

Coupled with the intrinsic limitations in ORR activity, the high cost of Ir remains a major barrier to its practical application [11, 12]. The recent surge in Ir market prices–now significantly surpassing those of Pt and Ru–has further constrained its widespread deployment. Alloying Ir with 3d transition metals (e.g., Co, Ni and Fe) in nanostructures represents a conventional yet effective strategy to modulate the electronic structure of Ir [13, 14, 15, 16]. This approach not only optimizes the adsorption energetics of oxygen intermediates and enhances intrinsic catalytic activity [17, 18], but also reduces noble metal consumption without compromising OER performance. For instance, Kwon and coworkers reported that in the Ir_3_CoO_*x*_–CMI catalyst, electron transfer from Co to Ir tunes the Ir *d*‐band center, achieving a low overpotential of 233 mV at 10 mA cm^−2^ [19]. However, while Ir‐based nanoalloys significantly enhance OER kinetics, they often remain insufficient for ORR. Consequently, relying solely on alloying is inadequate for achieving bifunctionality. A more comprehensive design is required to integrate distinct, yet synergistically coupled active centers that can independently optimize OER and ORR pathways.

Beyond alloying, anchoring Ir‐based nanomaterials onto heterogeneous substrates has become a primary strategy to optimize their structural stability and active center exposure [20, 21, 22]. In this context, the role of the support material could evolve from a physical scaffold that disperses active nanostructures into a functional component that actively regulate the local coordination environment and electronic structure of the active species [23, 24]. The metal–support interactions (MSI) not only suppress nanoparticle agglomeration but also tune the *d*‐band center of Ir sites [25, 26, 27], thereby optimizing the adsorption and desorption behavior of key reaction intermediates [28, 29]. Fe–N–C materials, characterized by porous carbon frameworks and atomically dispersed Fe–N_4_ coordination sites, have recently emerged as promising catalyst supports [30, 31, 32]. These materials not only stabilize metal nanostructures through MSI [33] but also contribute synergistically to ORR activity. Nevertheless, despite their proven effectiveness for ORR, Fe–N_4_ sites generally exhibit intrinsically poor activity toward the OER, limiting their direct application as bifunctional electrocatalysts [34, 35, 36]. This gap defines the core of our design: by integrating OER‐active Ir alloy nanostructures with ORR‐favored Fe–N_4_ sites into a hierarchical architecture, we can leverage their complementary strengths to achieve a bifunctional oxygen electrocatalyst.

Herein, we report the rational design and comprehensive evaluation of a hierarchical active‐site IrFe–N–C electrocatalyst with reduced Ir usage, in which IrFe alloy nanoparticles are uniformly anchored in a porous Fe–N–C matrix enriched with atomically dispersed Fe–N_4_ sites. The crystallographic structure and electronic properties of IrFe–N–C were systematically characterized using advanced electron microscopy and spectroscopic techniques. Evaluated in both half‐cell measurements and rechargeable Zn–air batteries, the catalyst demonstrates OER/ORR bifunctional activity and appreciable stability at a reduced Ir loading. Correlative structural–electrochemical analyses elucidate the complementary roles of the active sites, where IrFe nanoparticles preferentially catalyze the OER, while Fe–N_4_ moieties dominate the ORR. The MSI effectively stabilize both ultrafine nanoparticles and single‐atom sites under operating conditions. This work underscores a hybrid nano‐atomic catalysis strategy for achieving highly active, stable and cost‐efficient bifunctional electrocatalysts for advanced energy storage applications.

## 2 Results and Discussion

### 2.1 Synthesis and Conceptual Design of the IrFe–N–C

**FIGURE 1:**
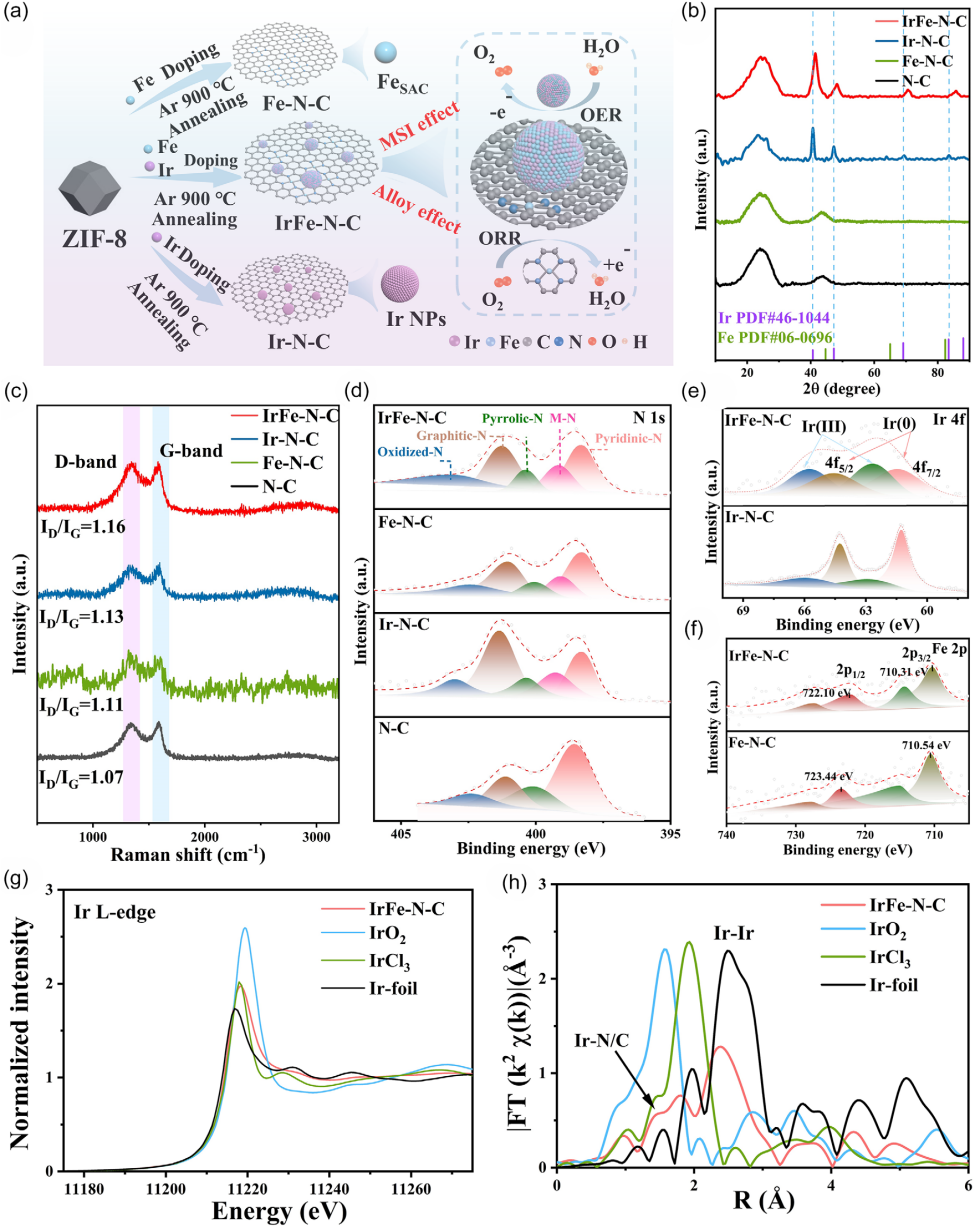
Synthesis, hierarchical active‐site architecture and physicochemical characterization of the IrFe–N–C and reference materials. (a) Schematic depiction showing the synthesis process and hierarchical active‐site architecture of the IrFe–N–C catalyst. (b) X‐ray diffraction (XRD) patterns of IrFe–N–C and the corresponding reference catalysts. (c) Raman spectra of IrFe–N–C, Ir–N–C, Fe–N–C, and N–C. (d–f) X‐ray photoelectron spectroscopy (XPS) spectra of IrFe–N–C and reference catalysts: (d) N 1s, (e) Ir 4f, (f) Fe 2p. (g) Ir L_3_‐edge XANES spectra of IrFe–N–C, IrCl_3_, IrO_2_, and Ir foil. (h) Fourier transformation of the EXAFS spectra in real (R) space.

As presented in Figure 1a, the IrFe–N–C was rationally designed with a hybrid hierarchical active‐site architecture by integrating IrFe alloy nanoparticles and atomically dispersed Fe–N_4_ sites within an N‐doped carbon framework. The catalyst was synthesized via a sequential doping–annealing strategy using ZIF‐8 as the template, in which Fe and Ir were successively incorporated to generate Fe–N–C, Ir–N–C and ultimately the IrFe–N–C material. Scanning electron microscopy (SEM) reveals that all precursor samples exhibit uniform particle dimensions and distinct dodecahedral morphologies (Figures S1 and S2). This hierarchical architecture synergistically combines alloying effects with MSI, thereby enabling cooperative catalytic behavior toward both the OER and ORR.

### 2.2 Physicochemical Characterizations of Prepared Electrocatalysts

First, X‐ray diffraction (XRD) was employed to identify the crystalline phases of a series of metal (M)–N–C catalysts. The Fe–N–C sample exhibits diffraction features nearly identical to those of N–C, indicating that the Fe species are highly dispersed and do not form detectable crystalline phases. In contrast, the Ir–N–C sample displays two characteristic diffraction peaks at approximately 40.66° and 47.30°, which can be indexed to the (111) and (200) planes of Ir, respectively [37, 38]. The Ir–N–C sample was further investigated by high‐angle annular dark‐field scanning transmission electron microscopy (HAADF‐STEM) (Figure S3), which enabled direct visualization of its morphology and microstructure, unambiguously confirming the presence of Ir nanoparticles. Upon incorporation of Fe, the diffraction peaks of IrFe–N–C exhibit an evident positive offset relative to those of Ir–N–C, indicative of lattice contraction arising from Ir–Fe alloy formation (Figure 1b) [28].

The Raman spectra of the M–N–C samples display the characteristic D and G bands of N‐doped carbon, as shown in Figure 1c. The intensity ratio (I_D_/I_G_) of IrFe–N–C (1.16) is greater than that observed for Ir–N–C (1.13), Fe–N–C (1.11) and N–C (1.07), indicating that the incorporation of metal coordination increases the defect density and the density of edge sites in the material. Notably, the nearly unchanged peak positions suggest that the overall graphitic framework remains well preserved. The elevated I_D_/I_G_ ratio of IrFe–N–C can be attributed to the local defects introduced by N doping and metal‐N coordination. The synergistic structure of the intact graphitic framework and local defects is beneficial for anchoring IrFe nanoparticles and stabilizing Fe–N_4_ moieties [39, 40].

The electronic structure of the IrFe–N–C catalyst was probed in depth by X‐ray photoelectron spectroscopy (XPS), as shown in Figure 1d–f. In the N 1s spectrum (Figure 1d), IrFe–N–C exhibits distinct components corresponding to pyridinic N (398.3 eV), metal‐coordinated N (M–N, 399.2 eV), pyrrolic N (400.3 eV), graphitic N (401.3 eV) and oxidized N (403.0 eV), in line with the characteristic features of Fe–N–C catalysts [41, 42]. Notably, pyridinic N and Fe–N coordination have been widely recognized as key structural motifs for the formation of Fe–N_4_ active sites [43, 44]. The pronounced presence of M–N species in the Ir(Fe)–N–C samples further indicates MSI between the Ir(Fe) nanoparticles and the N‐doped carbon framework. As shown in Figure 1e, the Ir 4f core‐level peaks of IrFe–N–C exhibit a positive binding‐energy shift relative to those of Ir–N–C, suggesting a modified electronic structure of Ir arising from interfacial charge redistribution induced by interactions with the Fe–N–C support. These observations collectively point to strengthened metal–support coupling in the hybrid system [45, 46, 47]. Meanwhile, the presence of Fe–N_4_ moieties can induce local electron deficiency in the carbon matrix, which is expected to promote stronger anchoring of IrFe nanoparticles and facilitate interfacial electron transfer. Further insights are provided by the Fe 2p XPS spectra (Figure 1f). The Fe 2p_3/2_ peak of IrFe–N–C appears at approximately 710.5 eV, comparable to that of Fe–N–C. However, IrFe–N–C exhibits a lower binding energy for the Fe 2p_1/2_ peak, indicating that the introduction of Ir alters the electronic structure of Fe through Ir–Fe alloying.

Beyond the aforementioned structural and surface characterization, the local electronic structure and coordination environment were further elucidated by synchrotron X‐ray absorption spectroscopy (XAS). As shown in Figure 1g, the Ir L_3_‐edge XANES spectrum of IrFe–N–C exhibits a significantly higher white line intensity compared to Ir foil. This observation indicates an increased density of unoccupied Ir 5d states, revealing a distinct electronic redistribution at the catalyst interface. Such electronic modulation serves as direct evidence of the MSI between the IrFe and the nitrogen‐doped carbon matrix. In the corresponding Fourier‐transformed (FT) EXAFS spectrum (Figure 1h), the dominant scattering peak at ˜ 2.4 Å corresponds to the metallic Ir–M (M = Ir, Fe) coordination, confirming the formation of the IrFe alloy phase [48, 49].

Fe K‐edge XAS and Wavelet Transform (WT) analysis were also conducted (refer to Figure S4a–d in the Supporting Information). The Fe EXAFS spectra clearly display dual coordination features representing both atomically dispersed Fe–N_4_ sites and metallic Fe—M bonds [50], while the WT plots resolve these distinct scattering centers in both R and k spaces [51]. These observations, consistent with the XPS results, rigorously substantiate the successful construction of a hierarchical nano‐atomic architecture, wherein the matrix not only provides specialized Fe–N_4_ ORR active centers but also effectively stabilizes the OER‐active IrFe nanoparticles through interfacial coupling.

In addition, inductively coupled plasma mass spectrometry (ICP‐MS) was used to quantify the metal loadings of the M–N–C materials, as summarized in Table S1. The Ir loading in IrFe–N–C is determined to be as low as 8.42 μg cm^−2^, which is substantially lower than that of most reported Ir‐based OER catalysts, underscoring the potential of this catalyst for practical and cost‐effective applications.

**FIGURE 2:**
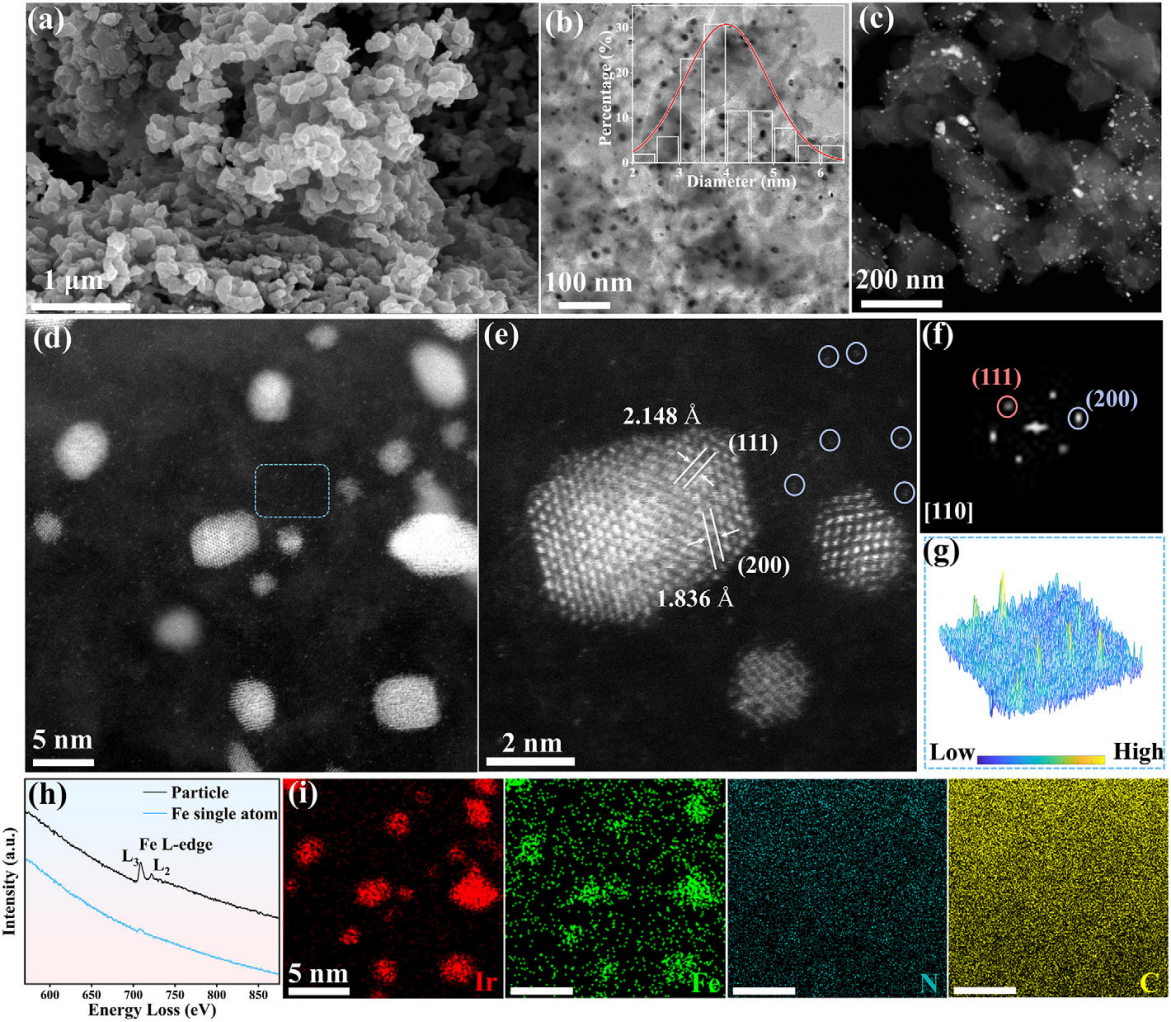
Electron microscopy characterization of the IrFe–N–C catalyst. (a) Scanning electron microscopy (SEM) image of IrFe–N–C. (b) Transmission electron microscopy (TEM) and (c) high‐angle annular dark‐field scanning transmission electron microscopy (HAADF‐STEM) images showing uniformly dispersed IrFe nanoparticles (insets: corresponding particle size distribution histograms). (d) Magnified HAADF‐STEM image revealing the coexistence of atomically dispersed single atoms and nanoparticles. (e) Atomic‐resolution HAADF‐STEM image of IrFe–N–C nanoparticles with resolved lattice fringes. (f) Corresponding fast Fourier transform (FFT) pattern of panel (e). (g) Three‐dimensional atomic topography image of the highlighted region in panel (d). (h) Corresponding electron energy‐loss spectroscopy (EELS) analysis in panel (e), confirming the presence of Fe single atoms in the IrFe–N–C electrocatalyst. (i) Elemental mappings acquired from panel (d).

Figure 2a presents an SEM image of IrFe–N–C, showing that the catalyst largely retains the polyhedral morphology of the precursor. Transmission electron microscopy (TEM) and HAADF‐STEM further investigated the microstructure. As shown in the low‐magnification bright‐field TEM image (Figure 2b), the IrFe nanoparticles exhibit a small average particle size of approximately 3.75 nm, as confirmed by statistical analysis. The corresponding HAADF‐STEM image (Figure 2c) reveals that the nanoparticles are uniformly dispersed on the Fe–N–C support. A magnified HAADF‐STEM image (Figure 2d) further confirms the coexistence of ultrafine nanoparticles and atomically dispersed single atoms on the carbon matrix. In Figure 2e, lattice fringes with spacings of 1.83 Å and 2.14 Å are observed, which can be indexed to the {200} and {111} planes of metallic Ir (PDF No. 46–1044), respectively. These slightly contracted d‐spacings, relative to pristine Ir, are consistent with the XRD results and provide direct evidence for Ir–Fe alloy formation. The corresponding fast Fourier transform (FFT) pattern (Figure 2f), acquired from the selected region in Figure 2e, further confirms a face‐centered cubic crystal structure with a [110] zone‐axis orientation. Moreover, a three‐dimensional intensity profile extracted from the blue dashed region in Figure 2d (Figure 2g) intuitively verifies the presence of isolated single atoms. A similar analysis was performed for the blue dashed region in Figure S5a and the resulting three‐dimensional intensity profile (Figure S5b) further corroborates the coexistence of nanoparticles and single atoms based on intensity contrast. As shown in Figure 2h, the electron energy‐loss spectroscopy (EELS) spectrum acquired from a representative nanoparticle exhibits a pronounced Fe L‐edge signal. The Ir signal is not detected due to the high energy‐loss threshold of the Ir M‐edge (approximately 2040 eV). Notably, atomic‐scale EELS collected from the region marked by the blue dashed circle in Figure 2e reveals a weak but discernible Fe L‐edge signal, confirming the atomically dispersed Fe species on the support [52]. The corresponding local magnified EELS is shown in Figure S6. Elemental mappings obtained from energy‐dispersive X‐ray spectroscopy (EDS) further show that Ir and Fe are homogeneously distributed within the nanoparticles, while Fe signals are also broadly dispersed over the N–C support (Figure 2i). Collectively, these results demonstrate that the IrFe–N–C catalyst consists of uniformly dispersed IrFe alloy nanoparticles anchored on a Fe–N–C matrix, accompanied by atomically dispersed Fe species embedded within the carbon framework.

### 2.3 Electrocatalytic Performance for OER

**FIGURE 3:**
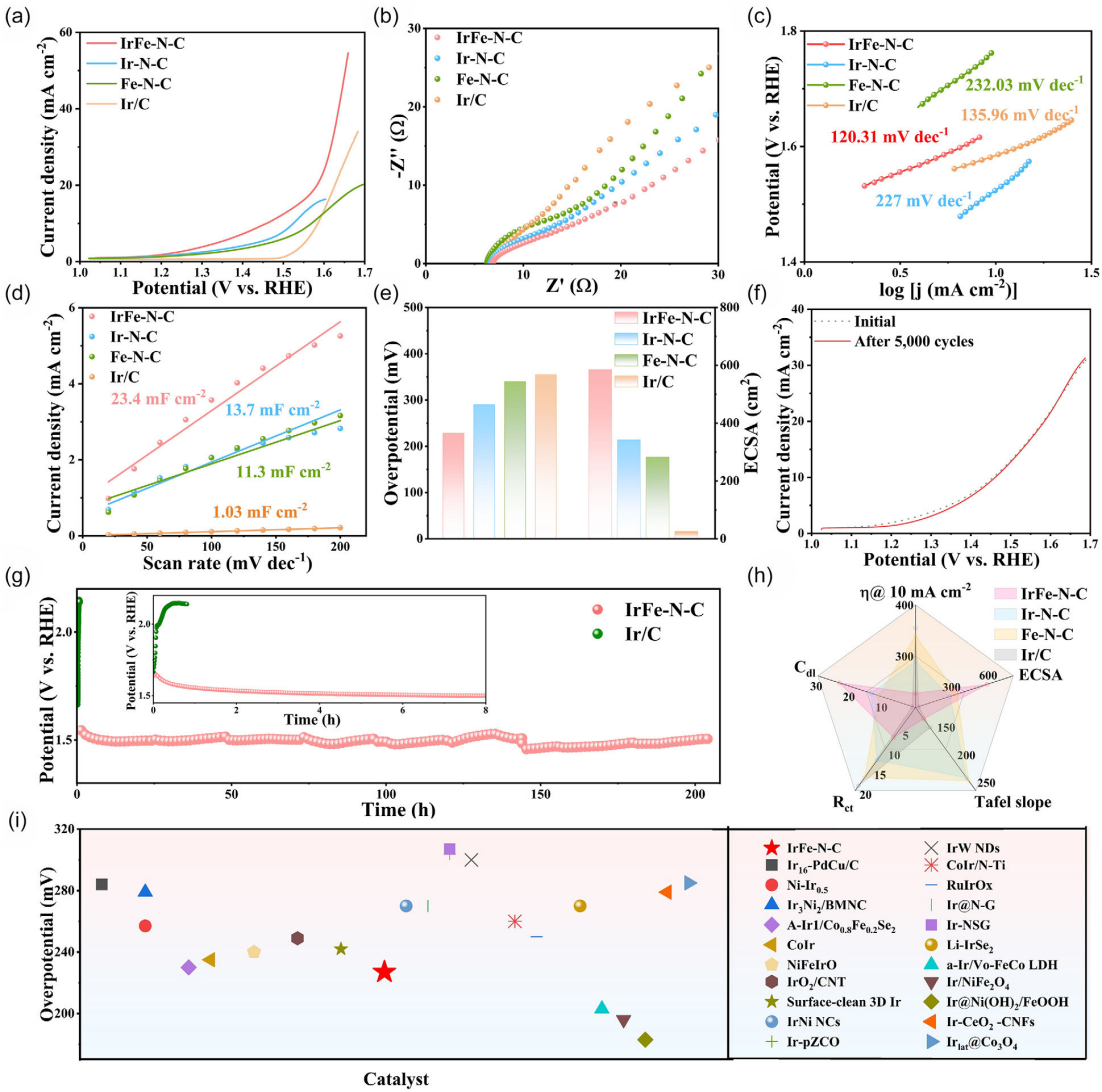
Oxygen evolution reaction (OER) electrocatalytic activity of IrFe–N–C and reference catalysts. (a) OER LSV profiles were measured under the conditions of 5 mV s^−1^ in 1.0 M KOH. (b) Electrochemical impedance spectroscopy (EIS) Nyquist plots. (c) Corresponding Tafel plots derived from the polarization curves. (d) Double‐layer capacitance (*C*_dl_) extracted from cyclic voltammetry measurements. (e) Comparison of overpotentials and electrochemically active surface areas (ECSA) for different catalysts. (f) Linear sweep voltammetry (LSV) curves recorded for IrFe–N–C in its initial and post‐durability test states. (g) Chronopotentiometric stability profiles of IrFe–N–C and reference materials measured applying a constant current of 10 mA cm^−2^ in 1.0 M KOH. (h) Radar chart summarizing key OER performance metrics. (i) Comparison of overpotentials at 10 mA cm^−2^ for IrFe–N–C and previously reported state‐of‐the‐art OER catalysts (references listed in Table S2).

To evaluate the OER performance, comprehensive electrochemical measurements were implemented for IrFe–N–C and the reference catalysts (Fe–N–C, Ir–N–C and commercial Ir/C) in 1.0 M KOH. Figure 3a presents the linear sweep voltammetry (LSV) curves of the catalysts under alkaline conditions. Among all samples, IrFe–N–C delivers the lowest overpotential of 227 mV at a current density of 10 mA cm^−2^, markedly outperforming Ir–N–C (290 mV), Fe–N–C (340 mV) and commercial Ir/C (355 mV). To better understand the subtle current change in the lower‐potential region of Figure 3a, online differential electrochemical mass spectrometry (DEMS) was conducted to qualitatively monitor the volatile products during the anodic sweep. As shown in Figure S7, the DEMS detects both O_2_ and CO_2_ signals during the forward scan. The emergence of the CO_2_ signal between 1.3 V and 1.5 V indicates that carbon‐support corrosion occurs within this initial potential window [53]. Given that carbon oxidation can proceed through the formation of surface oxygenated species prior to complete gasification, a portion of the anodic current in this pre‐OER region 1.1–1.5 V likely stems from carbon support modification rather than pure OER. Importantly, as the potential drives further into the OER regime, the O_2_ signal exhibits a sharp, exponential dynamic increase, suggesting that the ongoing anodic current is successfully dominated by the OER. Additionally, the higher low‐potential current density of IrFe–N–C compared to Fe–N–C (Figure 3a) could be attributed to pre‐onset OER Faradaic processes, implies that carbon corrosion might be suppressed owing to the OER activity of the IrFe–N–C catalyst [54, 55, 56]. These qualitative DEMS results together with the LSV profiles clarify the potential‐dependent evolution pathways. The OER polarization curve was normalized using electrochemically active surface area (ECSA) (Figure S8) and IrFe–N–C still showed an advantage [57]. Electrochemical impedance spectroscopy (EIS, Figure 3b) reveals that IrFe–N–C exhibits the smallest charge–transfer resistance (*R*_ct_), indicative of more efficient interfacial charge transport. Consistently, the Tafel plot shown in Figure 3c displays the lowest Tafel slope for IrFe–N–C, further confirming its favorable OER kinetics. Cyclic voltammetry (CV) measurements were conducted to estimate the ECSA via extraction of the double‐layer capacitance (*C*_dl_) (Figure S9). As shown in Figure 3d, IrFe–N–C exhibits the highest *C*_dl_ value of 23.4 mF cm^−2^, corresponding to an ECSA of approximately 600 cm^2^ (Figure 3e). This large ECSA reflects the abundance of accessible active sites afforded by the ultrafine and irregularly shaped IrFe nanoparticles. The intrinsic catalytic activity was further quantified by mass activity (MA) and turnover frequency (TOF) at an overpotential of 300 mV (Figures S10 and S11). Consequently, the MA of IrFe–N–C surpasses that of commercial Ir/C by a factor of 5.14, and its TOF demonstrates a 3.7‐fold improvement. Based on the above discussion, the competitive OER performance of IrFe–N–C is the result of the synergistic effect of increased active sites and enhanced intrinsic activity [58].

The stability of the IrFe–N–C catalyst was evaluated using two complementary tests. First, an accelerated durability test (ADT) consisting of 5,000 CV cycles was performed. As evidenced by Figure 3f, IrFe–N–C exhibits negligible performance degradation, with the overpotential at 10 mA cm^−2^ increasing by only 2 mV. Post‐test morphological analysis reveals that the IrFe nanoparticles remain uniformly dispersed on the carbon framework, whereas commercial Ir/C suffers from severe nanoparticle agglomeration (Figure S12). This observation is consistent with previously reported deactivation mechanisms associated with support corrosion under alkaline conditions. In addition, catalytic stability was tested at 10 mA cm^−2^ via chronopotentiometry (Figure 3g). IrFe–N–C demonstrates favorable OER stability for over 200 h with minimal performance decay, in stark contrast to Ir/C, which undergoes rapid deactivation within only a few hours. HAADF‐STEM characterization after the durability test (Figure S13) confirms that both the IrFe nanoparticles and the atomically dispersed metal species are well preserved, indicating robust anchoring to the Fe–N–C support. Notably, the nanoparticles retain a uniform average size of approximately 3.5 nm, very close to their initial dimensions, further verifying the structural robustness of the catalyst under prolonged alkaline OER operation. This favorable stability can be attributed to MSI, which effectively suppresses nanoparticle degradation and agglomeration. A radar chart summarizing key performance metrics, including overpotential at 10 mA cm^−2^ (*η*_10_), Tafel slope, ECSA, *R*_ct_ and *C*_dl_, is presented in Figure 3h. IrFe–N–C exhibits the most balanced and superior overall performance among all evaluated catalysts. Furthermore, comparison with established benchmarks in OER catalysis (Figure 3i and Table S2) underscores the optimized performance of IrFe–N–C. It also demonstrates good stability when compared with the state‐of‐the‐art Ir‐based catalysts (Table S3).

### 2.4 Electrocatalytic Performance for ORR

**FIGURE 4:**
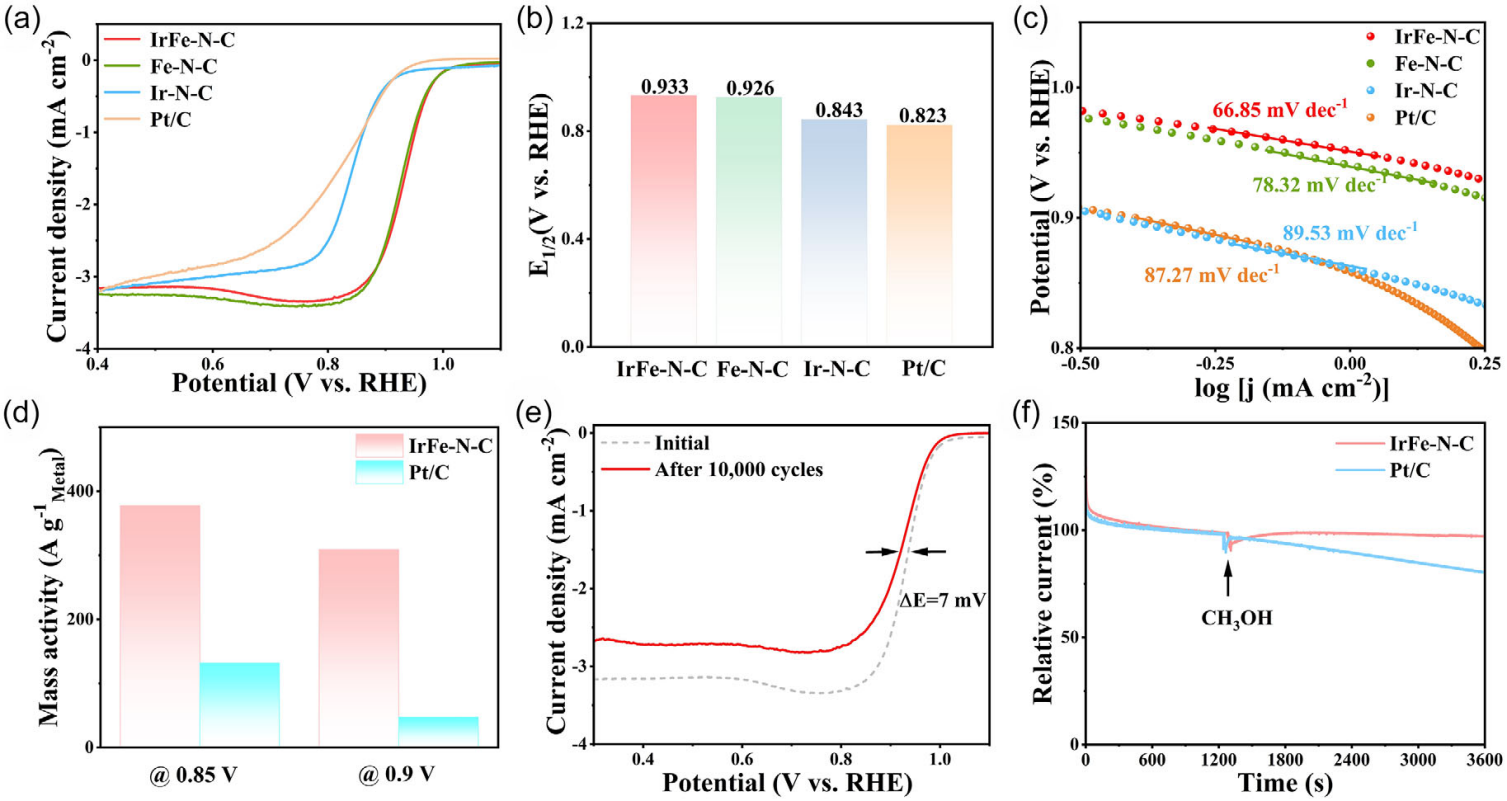
Oxygen reduction reaction (ORR) electrocatalytic activity of IrFe–N–C and reference catalysts. (a) LSV curves recorded in O_2_‐saturated 1.0 M KOH at a scan rate of 10 mV s^−1^ and a rotation speed of 1600 rpm. (b) Half‐wave potentials (*E*_1/2_) of various catalysts measured in 1.0 M KOH. (c) Corresponding Tafel plots derived from the LSV curves. (d) ORR mass activities of IrFe–N–C and commercial Pt/C. (e) LSV curves of IrFe–N–C before and after the durability test. (f) Chronoamperometric curves for commercial Pt/C and IrFe–N–C catalysts were recorded at 0.6 V versus RHE upon injecting 3.0 M methanol into 1.0 M KOH.

The ORR activity of IrFe–N–C was evaluated to elucidate its OER–ORR bifunctionality. Electrochemical measurements were carried out using a rotating disk electrode (RDE) in a three‐electrode configuration with 1.0 M KOH as the electrolyte. In O_2_‐saturated solution, ORR polarization curves were recorded by LSV at a rotation rate of 1600 rpm. As shown in Figure 4a,b, IrFe–N–C exhibits superior ORR performance, delivering an onset potential (*E*_on_) of 1.02 V and a half‐wave potential (*E*_1/2_) of 0.933 V versus the reversible hydrogen electrode (RHE). The comparable *E*_1/2_ values of IrFe–N–C and Fe–N–C indicate that Fe–N_4_ moieties serve as the primary ORR active centers in the hybrid catalyst. The ORR kinetics of IrFe–N–C are further corroborated by its smallest Tafel slope of 63 mV dec^−1^, which is significantly lower than those of Fe–N–C (78.32 mV dec^−1^), Ir–N–C (89.53 mV dec^−1^) and commercial Pt/C (87.27 mV dec^−1^) (Figure 4c). This enhanced kinetic behavior suggests that synergistic interactions between Ir and Fe are central to enhancing ORR performance. Additionally, the *C*_dl_ of IrFe–N–C, reflecting its electrochemically active surface area, is greater than that of Pt/C (Figure S14), indicating that the coexistence of dispersed nanoparticles and isolated Fe–N_4_ sites provides a greater density of accessible active sites. In addition, the ECSA of Pt/C was determined via the hydrogen adsorption–desorption method (Figure S15). Notably, at 0.85 and 0.90 V versus RHE, the MA of IrFe–N–C reached 377.6 and 308.7 A g^−1^_Metal_, respectively (Figure 4d), corresponding to enhancements of 2.86‐fold and 6.52‐fold relative to commercial Pt/C. These results underscore the ORR activity and noble‐metal utilization efficiency of the IrFe–N–C catalyst. Moreover, benchmarking our catalyst with the representative non‐noble NiFe oxyhydroxide@Fe–N–C systems (Table S4), although some NiFe‐based alternatives exhibit superior OER metrics, the IrFe–N–C delivers more balanced bifunctional kinetics that outperform most reported benchmarks, despite the cost advantage of NiFe alternatives. Furthermore, the Ir loading in this work was minimized to a low level, effectively curtailing the noble‐metal material cost.

Beyond catalytic activity, stability and ORR selectivity are critical metrics for practical applications. An ADT was performed at a scan rate of 100 mV s^−1^ over a potential window of 0.6–1.0 V versus RHE. After 10,000 cycles, the *E*_1/2_ of IrFe–N–C in 1.0 M KOH exhibits a negligible negative shift of only 7 mV (Figure 4e), whereas commercial Pt/C undergoes substantial performance degradation (Figure S16), highlighting the favorable stability of IrFe–N–C. Post‐ADT TEM analysis further confirms that IrFe–N–C retains its original particle size distribution, uniform dispersion and hybrid structural configuration. In contrast, commercial Pt/C suffers from severe nanoparticle agglomeration and morphological collapse (Figure S17). In addition, the ORR selectivity of IrFe–N–C was evaluated *via* a methanol tolerance test. As shown in Figure 4f, the injection of 3.0 M methanol into the electrolyte leads to a sharp decrease in current density for commercial Pt/C, reflecting its poor selectivity toward ORR. In sharp contrast, IrFe–N–C exhibits almost negligible current attenuation, demonstrating favorable methanol tolerance and highlighting its promising ORR selectivity for electrochemical applications.

To further simulate the actual dual‐function working conditions, we conducted a 10,000 cycles ADT with the OER/ORR potential range switching back and forth. As shown in Figure S18, after continuous alternating cycles, the dual‐function potential difference Δ*E* of the catalyst increased from the initial 0.550 V to only 0.582 V and the overall performance degradation was only 5.8%. This result fully proves that IrFe–N–C can still maintain the dual‐function catalytic output under the potential cycling conditions of alternating ORR and OER.

### 2.5 Rechargeable Zn–Air Battery (ZAB) Performance of the Prepared Electrocatalyst

**FIGURE 5:**
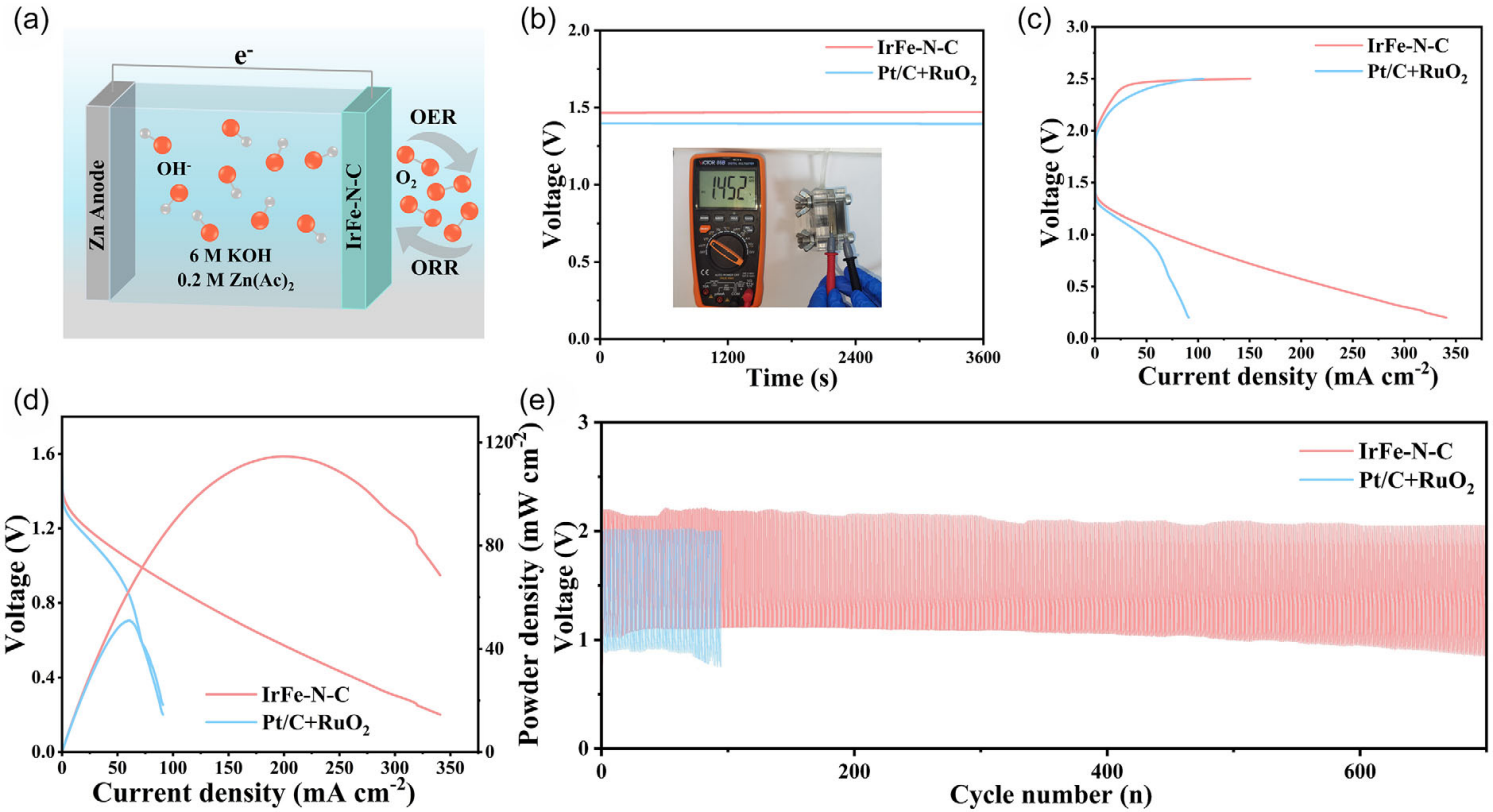
Zn–air battery test using IrFe–N–C and Pt/C + RuO_2_ catalysts. (a) Schematic diagram of the battery configuration. (b) Open‐circuit voltage (OCV) profiles. (c) Charge and discharge polarization curves. (d) Polarization and corresponding power density curves for Zn–air batteries. (e) The cycling durability of the two‐electrode rechargeable Zn–air battery with the IrFe–N–C air cathode under a current density of 10 mA cm^−2^.

Given its promising OER–ORR bifunctional activity, the practical applicability of IrFe–N–C was further evaluated by assembling a rechargeable ZAB using IrFe–N–C as the air cathode and zinc foil as the anode. For comparison, a ZAB employing a Pt/C + RuO_2_ cathode was also constructed. The operating principle of the ZAB is schematically illustrated in Figure 5a. The IrFe–N–C‐based ZAB delivers a high open‐circuit voltage (OCV) of 1.452 V (Figure 5b), corresponding to approximately 88% of the theoretical maximum voltage (1.65 V) under alkaline conditions and exceeding the 1.392 V achieved by the benchmark Pt/C + RuO_2_ system [59, 60]. As shown in the charge–discharge polarization curves (Figure 5c), the ZAB equipped with the IrFe–N–C cathode demonstrates substantially lower polarization losses across the entire current density range, reflecting its superior ORR and OER kinetics. Meanwhile, the porous carbon framework constructs a continuous conductive network and efficient mass transport pathways, which facilitates rapid charge transfer and electrolyte diffusion by reducing activation polarization and concentration polarization [61, 62]. Consequently, the IrFe–N–C‐based ZAB achieves a peak power density of 114.5 mW cm^−2^, more than twice that of the Pt/C + RuO_2_ counterpart, which delivers only 51 mW cm^−2^ (Figure 5d). The cycling stability of the batteries was further evaluated through repeated charge–discharge measurements (Figure 5e). The IrFe–N–C‐based ZAB demonstrates fair stability, maintaining stable operation with negligible performance decay over 700 cycles. To further evaluate the durability under benchmark‐level operational limits, the test was extended up to 1,000 cycles, with the complete chronological voltage data compiled in Figure S19. These preliminary device‐level results exhibit a polarization broadening after 800 cycles. Such late‐stage degradation can be attributed to a multitude of factors, including anode shape deformation and electrolyte issues. Nevertheless, the IrFe–N–C system successfully completed the rigorous 1,000‐cycle test and significantly outperformed the commercial Pt/C+RuO_2_ counterpart (which failed before 100 cycles), demonstrating favorable durability for practical energy applications.

The IrFe–N–C catalyst demonstrates favorable ORR/OER bifunctional performance in both half‐cell electrochemical evaluations and assembled Zn–air batteries. Comprehensive structure–electrochemical correlations reveal that this dual activity originates from the hybrid, hierarchical architecture comprising IrFe alloy nanoparticles and atomically dispersed Fe–N_4_ sites, which preferentially catalyze the OER and ORR, respectively. Notably, the catalyst also demonstrates appreciable stability, despite the intrinsic instability typically associated with ultrafine nanoparticles and single‐atom species. This robustness arises from the MSI: the Fe–N_4_ moieties are firmly embedded within the N‐doped carbon matrix during pyrolysis, while high‐temperature IrFe alloying establishes interfacial bonding with the carbon support. Such synergistic anchoring effectively suppresses particle migration and coalescence, thereby preserving active‐site accessibility and ensuring sustained catalytic integrity under operating conditions.

## 3 Conclusions

In conclusion, this work reports a hierarchical active‐site IrFe–N–C electrocatalyst that integrates IrFe alloy nanoparticles and atomically dispersed Fe–N_4_ sites within a conductive N‐doped carbon framework. This hybrid architecture enables clear functional complementarity, in which IrFe nanoparticles efficiently catalyze the OER while Fe–N_4_ moieties promote the ORR, resulting in highly synergistic bifunctional activity. The porous N‐doped carbon matrix stabilizes both ultrafine nanoparticles and single‐atom sites through MSI. Benefiting from this rational design, IrFe–N–C achieves an OER overpotential of only 227 mV at 10 mA cm^−2^ and delivers a MA more than five times greater than that of commercial Ir/C, together with a 6.5‐fold enhancement in ORR MA relative to Pt/C, all at an ultralow Ir loading of ˜ 8.5 μg cm^−2^. Moreover, the catalyst exhibits appreciable stability in both half‐cell measurements and full‐cell ZAB configurations. Beyond its immediate performance metrics, this work highlights hierarchical active‐site engineering combined with rational support design as a general and effective strategy for developing advanced electrocatalysts for energy conversion and storage technologies.

## Funding

This work was supported by National Key Research and Development Program of China (Grants 2021YFA1400204, 2021YFA0718701), National Natural Science Foundation of China (Grants 12474022, 12004340, 12474021, 22102077), Natural Science Foundation of Henan Province (Grant 252300421209), Science and Technology Innovation Leading Talent Support Program of Henan Province (Grant 254000510036).

## Conflicts of Interest

The authors declare no conflicts of interest.

## Supporting information

Supplementary Material

## Data Availability

The data that support the findings of this study are available from the corresponding author upon reasonable request.
