# Supplementary material for: Hybrid Nano‐Atomic IrFe–N–C for Low‐Iridium Oxygen Electrocatalysis
Source: Small Sci. 2026 Jul 29;6(8):e70358. doi: 10.1002/smsc.70358 (PMC13431759; doi:10.1002/smsc.70358)
Supplement: Supplementary file 1 — Supplementary Material [file SMSC-6-e70358-s001.pdf]

Supporting Information

Hybrid Nano-Atomic IrFe–N–C for Low-Iridium Oxygen Electrocatalysis

Meng-Meng Liu[1,†], *Han Gao*[1,†,]*, *Shi-Yu Zhu*[1], *Meng Li*[2], *Xiao Ren*[1,3], *Yi-Fang Yuan*[1], *Wei-Jie Liu*[1],*Ling-Rui Wang*[1], *Tian-Yu Xia*[1], *Xiao-Lei Shi*[2], *Haizhong Guo*[1,3,]*, *Zhi-Gang Chen*[2,] *

[†]indicates authors are equally contributing: Meng-Meng Liu, Han Gao

[*]indicates the corresponding authors: Han Gao, Haizhong Guo, Zhi-Gang Chen

Affiliations

[1] *School of Physics, Zhengzhou University, Zhengzhou, 450001, China*

[2] *School of Chemistry and Physics, ARC Research Hub in Zero-emission Power Generation for Carbon Neutrality, and Centre for Materials Science, Queensland University of Technology, Brisbane, Queensland, Australia*

[3] *Institute of Quantum Materials and Physics, Henan Academy of Sciences, Zhengzhou,450046, China*

Han Gao: hgao@zzu.edu.cn

Haizhong Guo: hguo@zzu.edu.cn

Zhi-Gang Chen: zhigang.chen@qut.edu.au

1. Experimental Section

Reagents.

2-methylimidazole ($C_4H_6N_2$, Aladdin), zinc nitrate hexahydrate (Zn $(NO_3)_2$•$6H_2O$, Aladdin), methanol (CH_3OH, Scharlau), ethanol (C_2H_5OH, VWR), n-hexane Ferric chloride hexahydrate ($FeCl_3\cdot6H_2O$, Aladdin), sodium hexachloroiridium hydrate ($Na_3IrCl_6\cdot xH_2O$, SigmaAldrich), Nafion (117, Sigma, 5% by weight) and potassium hydroxide (KOH, Aladdin) 70%) were used for electrochemical measurement. Commercial Pt/C and commercial Ir/C were purchased from Shanghai Heshen and Sinero technology, respectively. All the materials were used without further treatment.

Synthesis of ZIF-8

In a typical synthesis, Zn $(NO)_3\cdot6H_2O$ (1.762 g) was dissolved in 120 mL of methanol and stirred till fully dissolved. Then, 2-methylimidazole (3.912 g) was added to 120 mL of methanol and vigorously stirred at room temperature for 6 hours. The obtained product was centrifuged and washed several times with methanol, and finally dried overnight in an oven at 60 °C.

Synthesis of IrFe–N–C, Fe–N–C, Ir–N–C and N–C

IrFe–N–C was synthesized by first dispersing 100 mg of ZIF-8 in 16 mL of hexane under ultrasonication for 1 h. Subsequently, 60 μL of $FeCl_3\cdot6H_2O$ (20 mg mL^{-1}) and 80 μL of $Na_3IrCl_6\cdot xH_2O$ (20 mg mL^{-1}) aqueous solutions were successively added dropwise under ultrasonication over 10 minutes. After stirring for 3 h, the impregnated solid (IrFe@ZIF-8) was collected by centrifugation, dried overnight at 60 °C, and finally annealed at 900 °C for 2 h under Ar flow to obtain the catalyst. For comparison, the reference catalysts Ir–N–C and Fe–N–C were prepared using same procedures but with 80 μL of $Na_3IrCl_6\cdot xH_2O$ or 60 μL of $FeCl_3\cdot6H_2O$ solution as the sole metal precursor, respectively. The pure N–C support was obtained by direct pyrolysis of ZIF-8 without metal loading.

Physicochemical characterization

Transmission electron microscopy (TEM) images were obtained on a Thermofisher Talos 200s microscope at an accelerating voltage of 200 kV. High resolution scanning transmission electron microscopy (HR-STEM) images and energy-dispersive X-ray

spectroscopy (EDS) were obtained on a JEOL NEOARM200 Cs-corrector (S)TEM at an accelerating voltage of 200 kV. The EELS was collected at a JEOL ARM300F Cs-corrector (S)TEM at an accelerating voltage of 300 kV. The crystalline phases of the sample are determined by X-ray diffraction analysis (XRD, PANalytical Empyrean), and X-ray photoelectron spectroscopy (XPS) were obtained on a Thermo Scientific K-Alpha. The X-ray absorption spectra were acquired at Shanghai Synchrotron Radiation Facility (SSRF, Shanghai, China). The types and contents of chemical elements in the samples were measured by inductively coupled plasma mass spectrometry (ICP-MS, Agilent 7800). High-pressure Raman spectra were acquired using a 532 nm laser (10 mW, MSL-U-532 nm, Cnilaser) coupled to a Raman spectrometer (HRS-500MS, Princeton Instruments) and a highly sensitive thermoelectrically cooled CCD detector (PIX-256E, Princeton Instruments).

Electrochemical measurements

All the measurements were carried out at room temperature on the CH Instruments 660E (Shanghai Chenhua 660E) electrochemical workstation, using a three-electrode electrochemical device with a rotating disk electrode (RDE) system. For ORR and OER measurements, the working electrode is glass carbon electrode (GCE) (diameter: 5 mm, area: 0.196 cm^{-2}), using saturated Ag/AgCl electrode and carbon rod as reference electrode and counter electrode, respectively. All the potentials were calibrated to reversible hydrogen electrodes (RHE) with reference to this formula:

$$\mathrm{E(vs.RHE)} = \mathrm{E(vs.Ag/AgCl\,)} + 0.197\ \mathrm{V} + 0.0591 * \mathrm{pH}.$$

First of all, a working electrode is prepared, 2 mg of catalyst is dissolved into a mixed solution of 245μL of deionized water, 245 μL of alcohol, 8 μL of Nafion, and sonicated for 30 min to form a homogeneous catalyst ink. The prepared catalyst ink is dropwise added to the surface of the glassy carbon electrode to form a thin film with a load density of 8.42 μg cm^{-2} Ir. Then, dried naturally at room temperature.The apparent OER activity was characterized using cyclic voltammetry (CV) at a scan rate of 10 mV s^{-1}. An iR-correction of 90 % was applied to compensate for the voltage drop between the reference and working electrodes, which was measured by a single-point high-frequency impedance test. The ORR performance of catalysts was

measured by linear sweep voltammetry from 1.1 to 0 V versus RHE in O_2-saturated 1.0 M KOH solutions with a scanning rate of 10 mV s^{-1}. The revolution speed was set to 1600 rpm.The ECSA of the catalyst sample is calculated from the double layer capacitance according to the following equation:

$$\mathrm{ECSA} = \mathrm{C_{dl}}/\mathrm{C_s},$$

where $\mathrm{C_s}$ is the specific capacitance of the sample, we use general specific capacitances of $\mathrm{C_s}$ = 0.04 mF cm^{-2} based on typical reported values. The double-layer capacitance (C_{dl}) of catalysts was estimated by performing CV in the potential range of 0 to 0.1 V versus SCE (non-Faradaic potential range) at different scan rates (v) of 20, 40, 60, 80, 100, 120, 140, 160, 180, and 200 mV s^{-1}, followed by extracting the slope from the resulting $|j_a - j_c|/2$ versus v plots (j_a and j_c represent the anodic and cathodic currents at −0.60 V vs SCE). The turnover frequency (TOF) was calculated based on equation:

$$\mathrm{TOF} = \mathrm{j} \times \mathrm{A}/(4 \times \mathrm{F} \times \mathrm{n})$$

$$n = \frac{m_{Ir}}{M_{Ir}}$$

where j is the current density, A is the electrode area, F is the Faraday constant (96,485 C mol^{-1}), and n is the number of Ir atom participating in the reaction, where m_{Ir} is the mass of Ir loaded on the electrode and, M_{Ir} is the molar mass of Ir. Mass activity (A g^{-1}_{Metal}) values were calculated from the electrocatalyst loading m and the measured current density j at overpotentials of 300 mV:

$$\text{Mass activity} = \frac{\mathrm{j}}{\mathrm{m}}.$$

The OER stability was assessed at a constant current density of 10 mA cm^{-2}. The ADT of ORR was performed in the potential range of 0.6−1.0 V versus RHE at 100 mV s^{-1} for continuous 10,000 cycles. Furthermore, the poison tolerance was investigated at 0.6 V versus RHE after introducing 3 M methanol in O_2-saturated 1.0 M KOH solution, using the RDE at a rotation rate of 1600 rpm.

Testing of Zinc−Air Battery

Zinc-air battery (ZAB) was prepared and evaluated at ambient conditions. The ZAB was composed of a Zn plate as the anode, a 6.0 M KOH and 0.2 M $ZnCl_2$ aqueous

solution as the electrolyte, and the IrFe−N−C catalysts as the cathode. The air electrode was prepared by drop-casting as-prepared catalyst ink onto hydrophobic carbon paper (Fuel Cell Store, effective area = 1 cm^2), yielding a catalyst loading of 1.0 mg cm^{-2}. For comparison, commercial Pt/C and RuO_2 mixed catalyst (mass ratio of 1:1) was also prepared by the same procedure to form. All battery performance tests were performed under an ambient atmosphere. The charge–discharge cycling tests of the rechargeable ZABs were performed at 10 mA cm^{-2} with 20 min per cycle (10 min for charging and 10 min for discharging) using the CS310X testing system.

2. Supplementary Figures and Tables

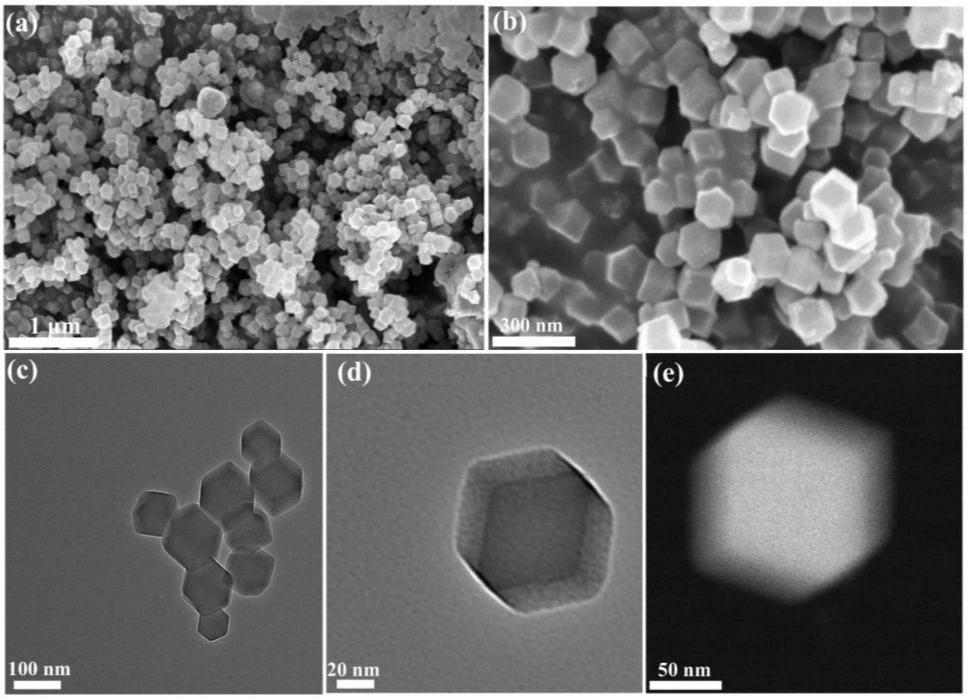

Figure S1. a,b) SEM and c-e) STEM images of ZIF-8 at different magnifications.

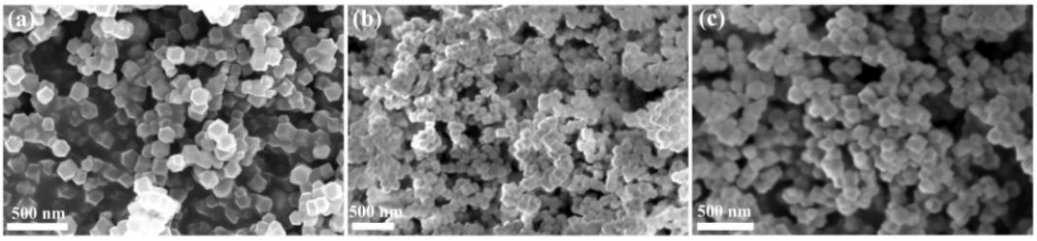

Figure S2. SEM images showing the morphology of a) Ir@ZIF-8, b) Fe@ZIF-8 and c) IrFe@ ZIF-8.

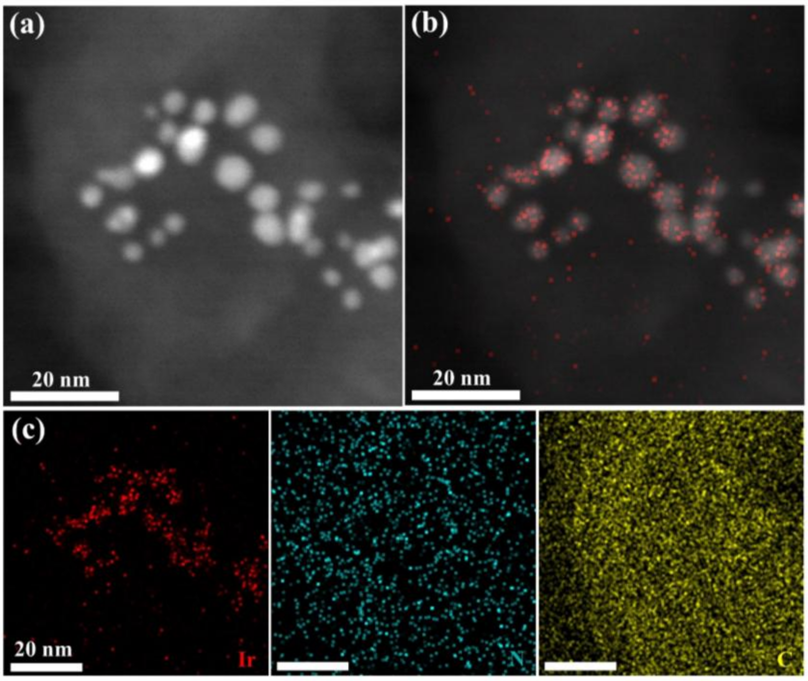

Figure S3. a) HAADF-STEM image and b,c) EDS mapping of Ir–N–C.

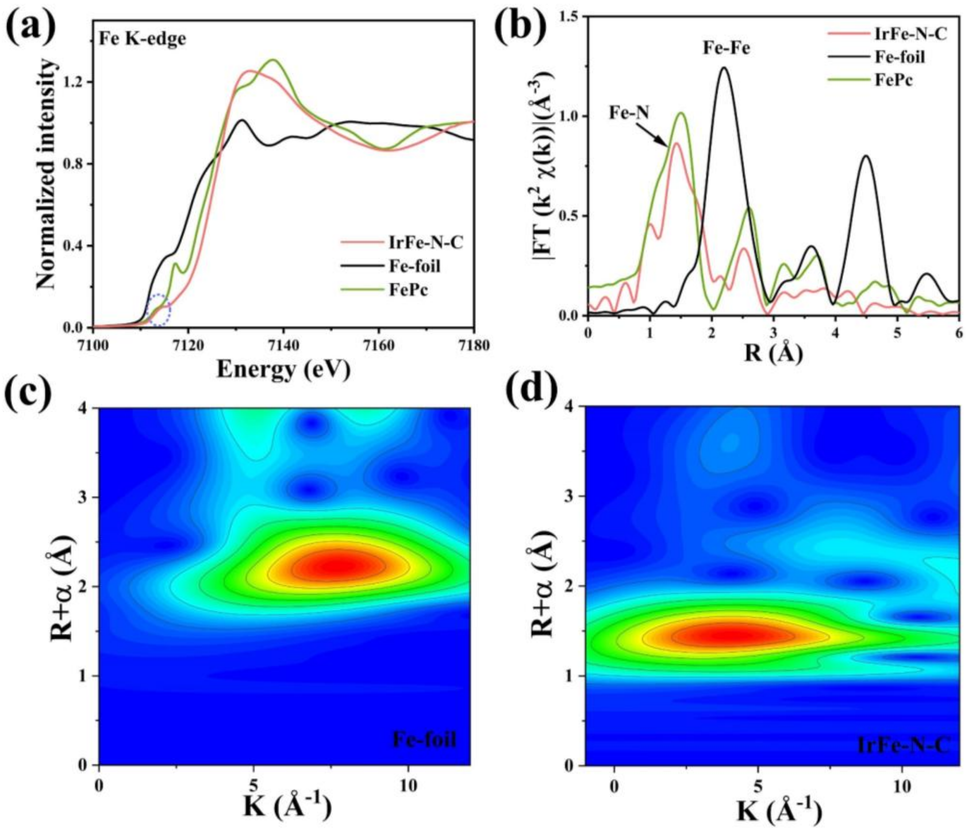

Figure S4. a) XANES and b) FT-EXAFS spectra of IrFe–N–C and reference materials at the Fe K-edge. Wavelet transform contours of the Fe K-edge from (c) Fe foil and (d) IrFe−N−C.

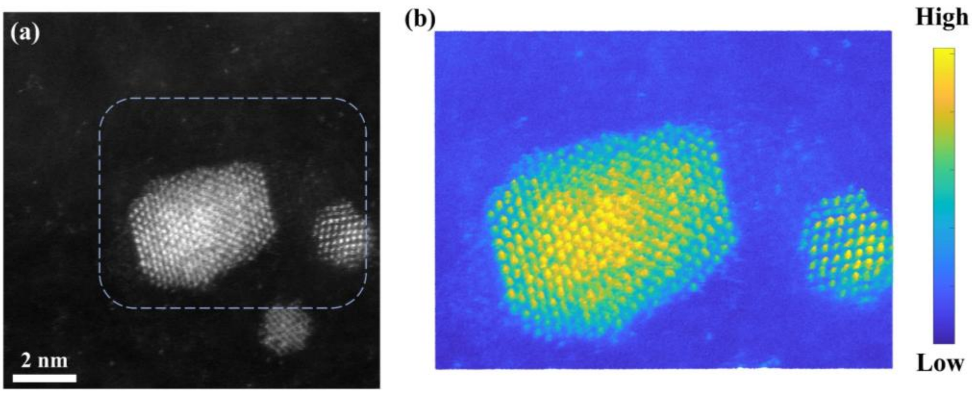

Figure S5. a) HAADF-STEM image of IrFe–N–C. b) 3D atomic topography image of the marked region in a).

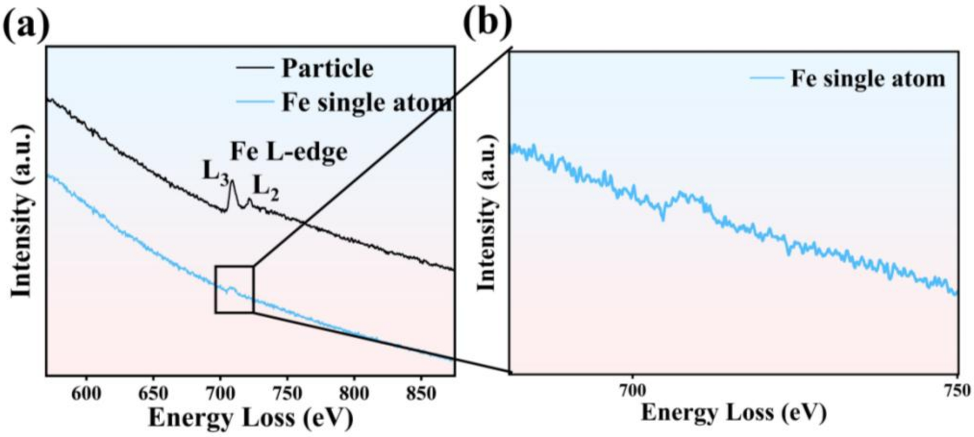

Figure S6. a,b) The corresponding local magnified image of EELS.

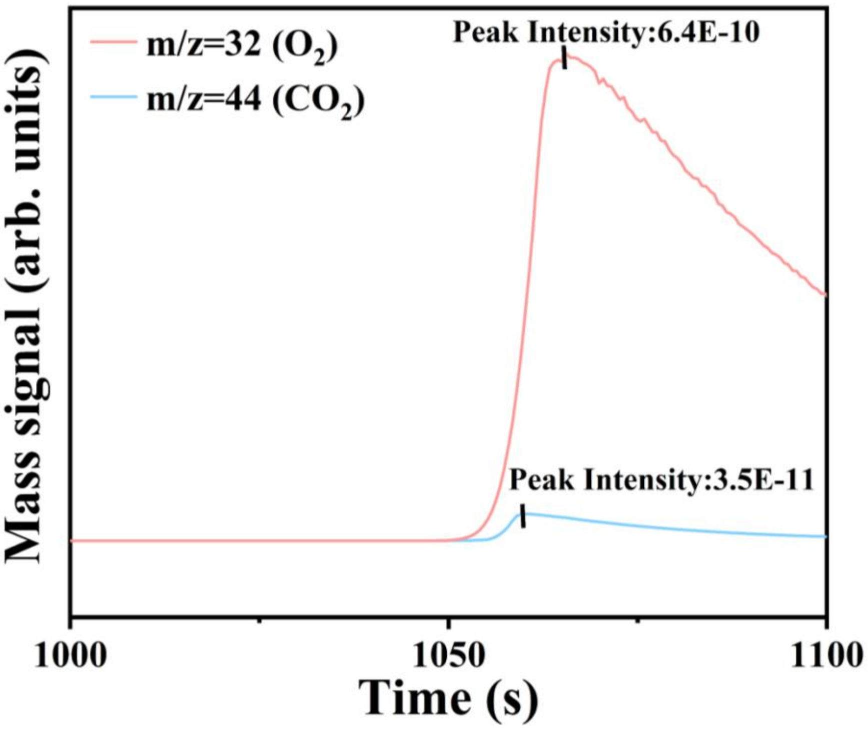

Figure S7. Differential electrochemical mass spectrometry (DEMS) signals of O_2 (m/z = 32) and CO_2 (m/z = 44) recorded during LSV scanning from 1.0 to 1.8 V vs. RHE.

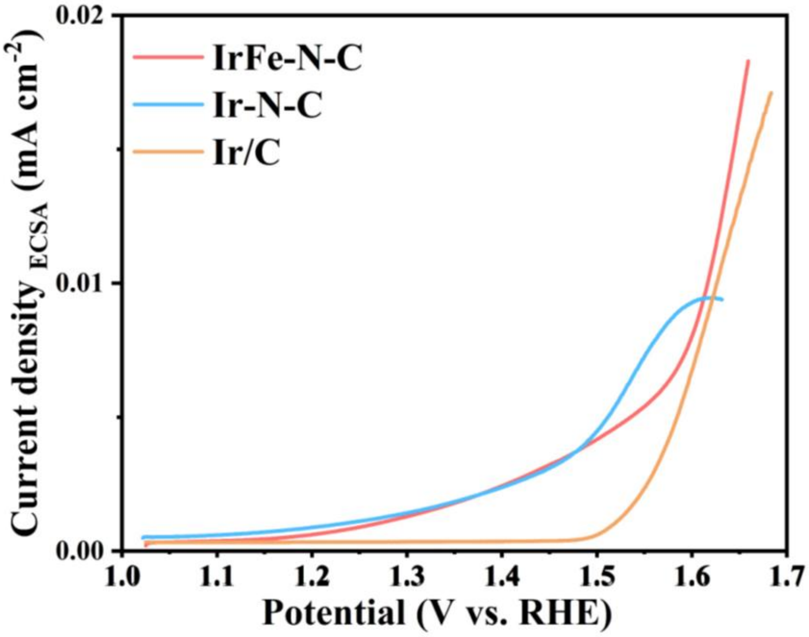

Figure S8. ECSA-normalized OER polarization curves of IrFe–N–C, Ir–N–C, and Ir/C.

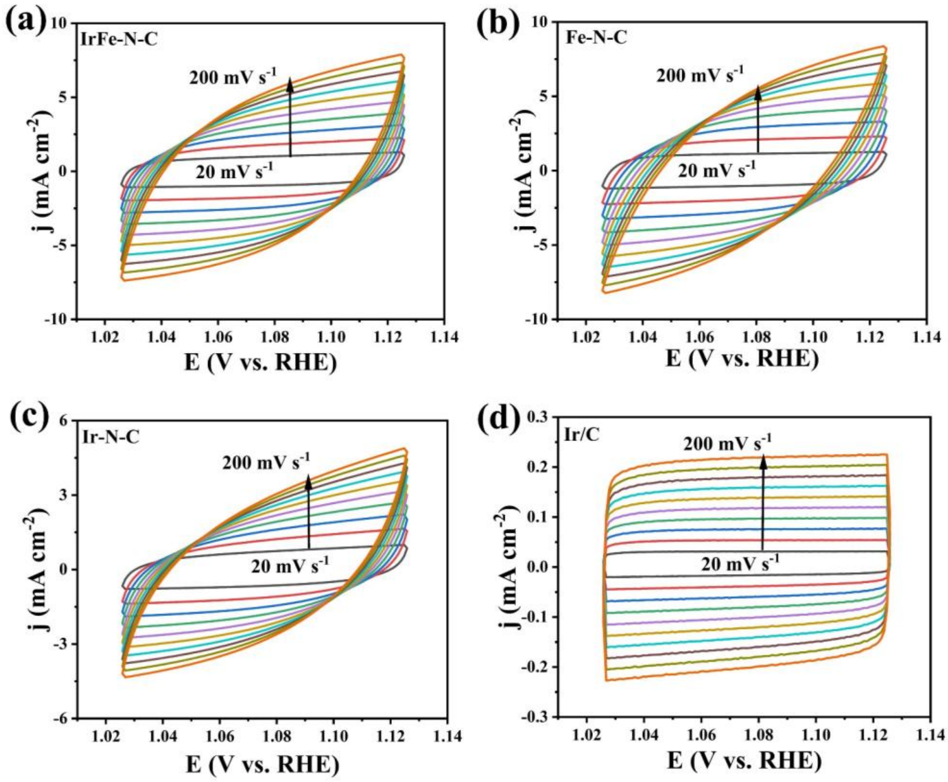

Figure S9. Electrochemical CV curves of a) IrFe–N–C, b) Fe–N–C, c) Ir–N–C, and d) Ir/C at different scan rate.

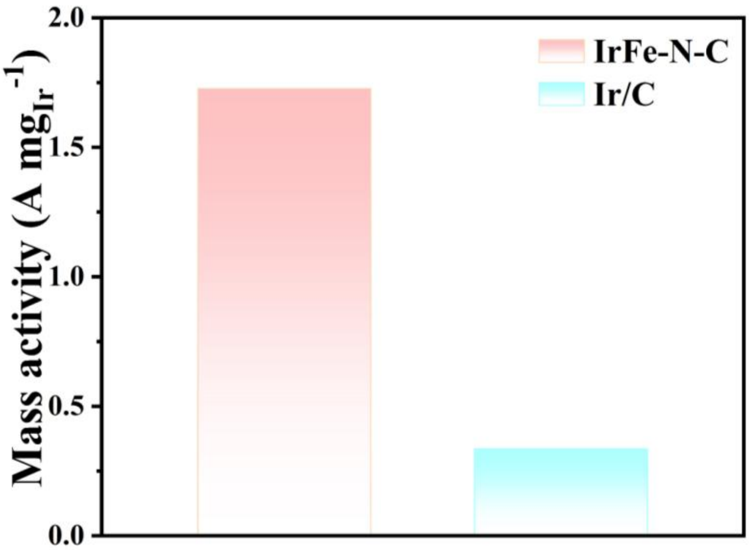

Figure S10. Mass activity (at 1.53 V_{RHE}) of IrFe–N–C and Ir/C.

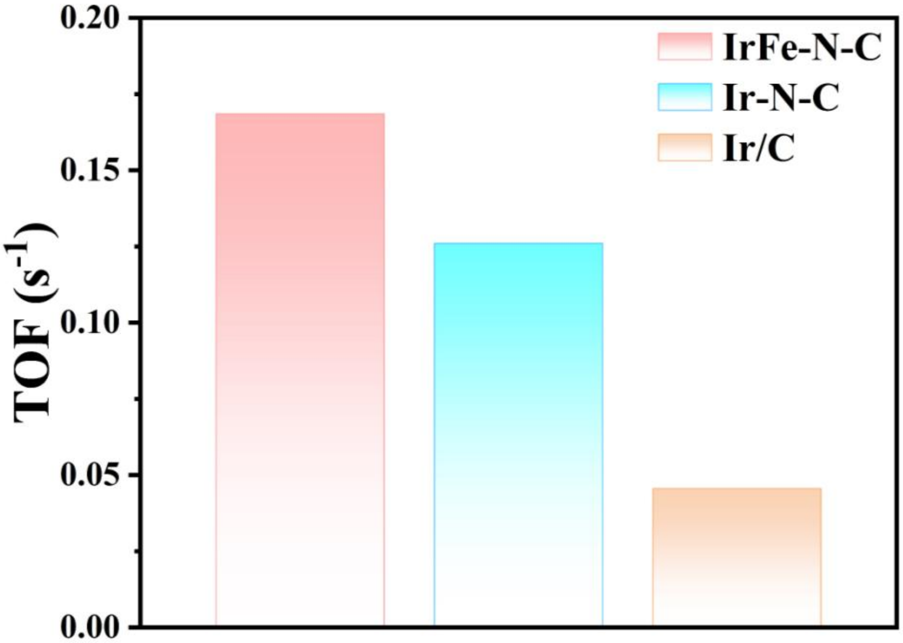

Figure S11. Ir TOF of IrFe–N–C, Ir–N–C and Ir/C at an overpotential of 300 mV.

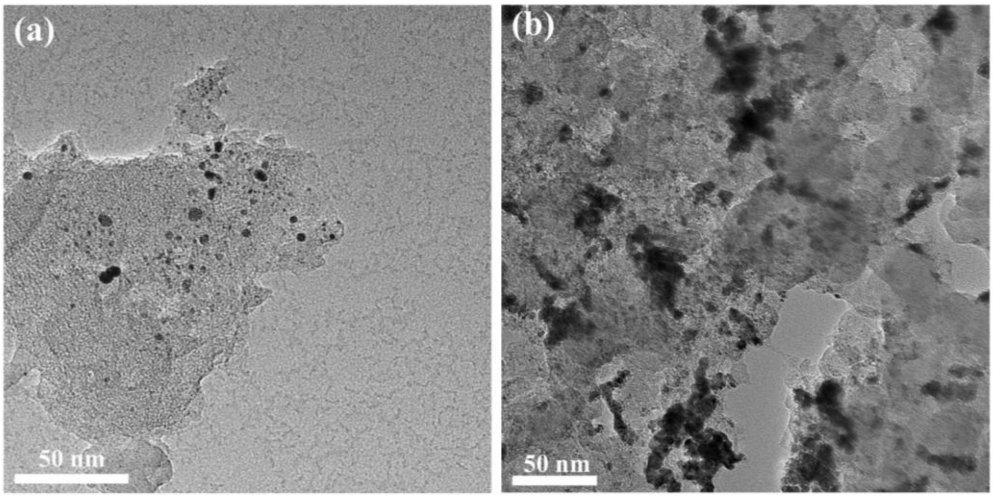

Figure S12. TEM images of a) IrFe–N–C, b) Ir/C after 5,000 cycles of ADT.

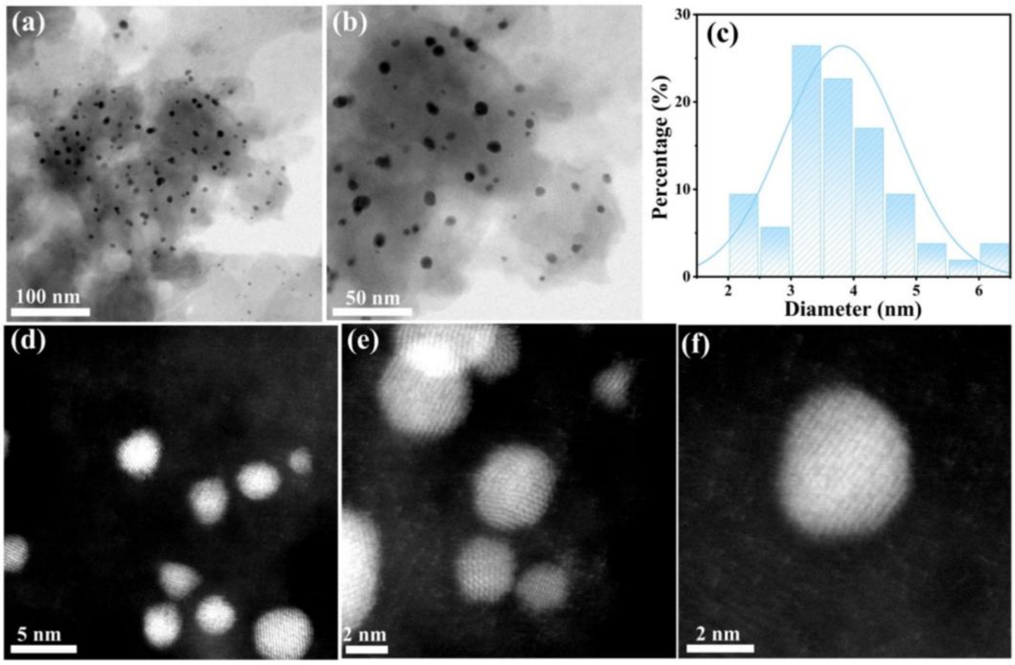

Figure S13. Electron microscopy characterization of IrFe–N–C after 200 hours of constant current chronopotentiometric testing. a,b) TEM images at different magnifications. c) The particle size distribution histogram in a) shows an average particle size of ≈3.5 nm. d-f) HAADF-STEM image of the IrFe–N–C sample.

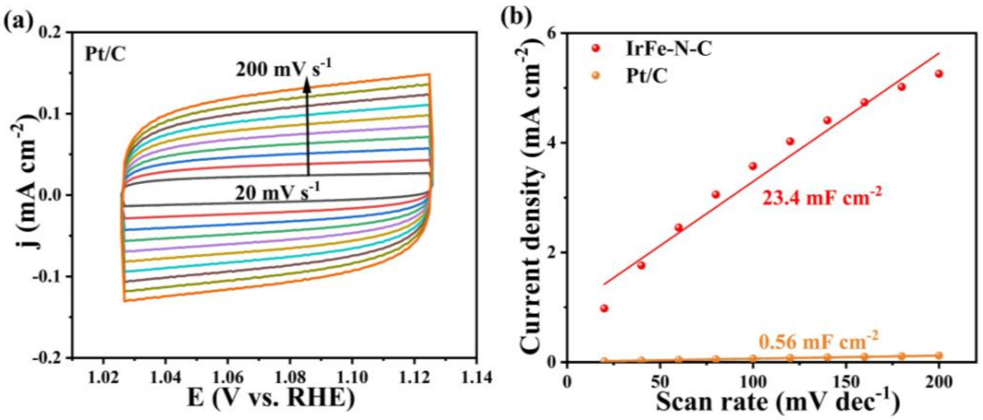

Figure S14. a) CV measurement of Pt/C at different scan rate. b) Double-layer capacitance of IrFe–N–C and Pt/C.

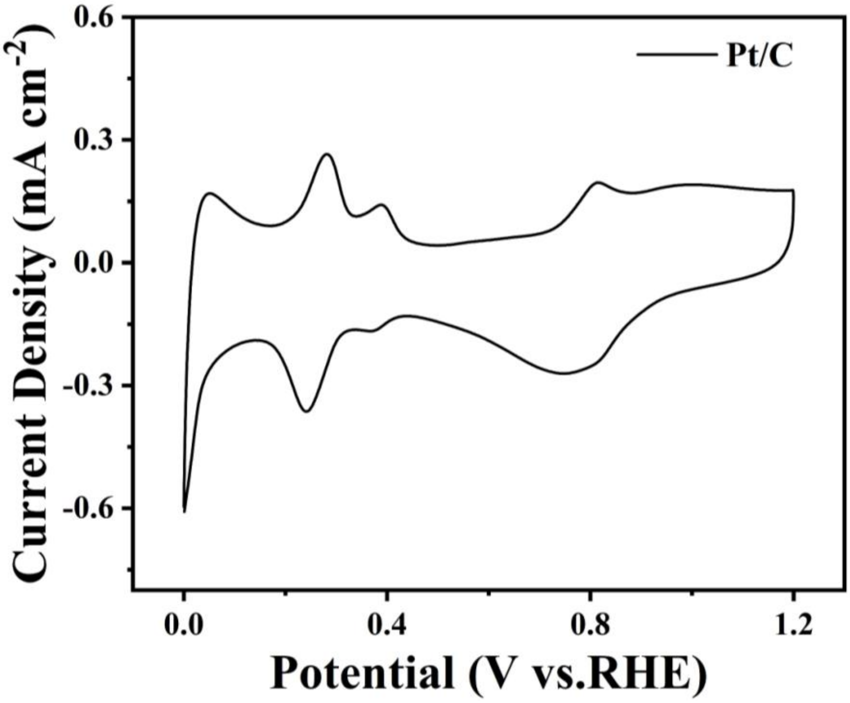

Figure S15. CV curves of Pt/C for hydrogen adsorption-desorption. The ECSA of Pt/C was calculated to be 59.8 $m^2\ g^{-1}_{Pt}$ (Pt loading: 9 μg cm^{-2}).

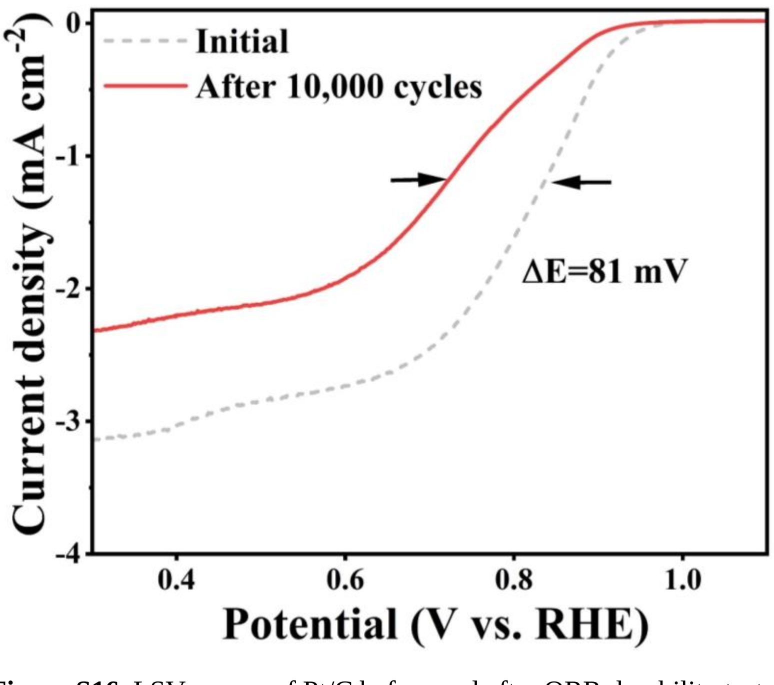

Figure S16. LSV curves of Pt/C before and after ORR durability test.

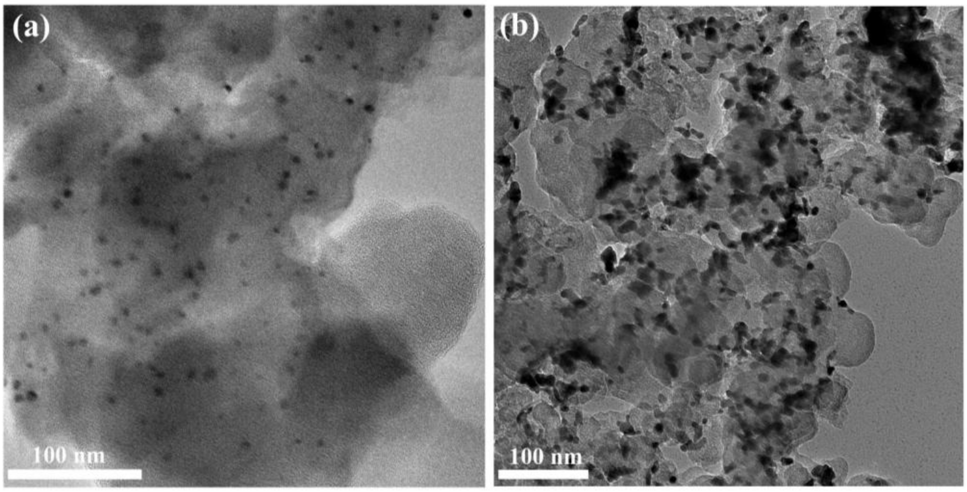

Figure S17. TEM images of a) IrFe–N–C, b) Pt/C after 10,000 cycles of ADT.

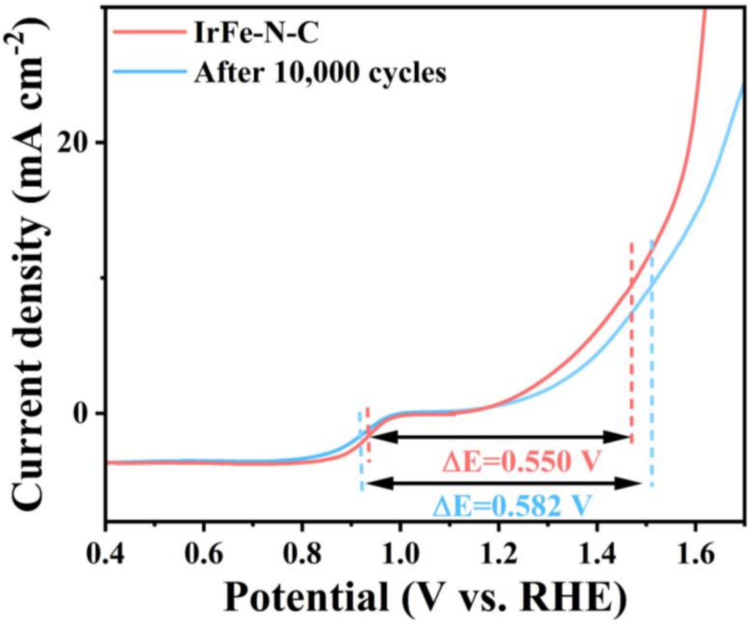

Figure S18. Bifunctional OER/ORR polarization curves of IrFe-N-C before and after 10,000 continuous potential cycles of ADT.

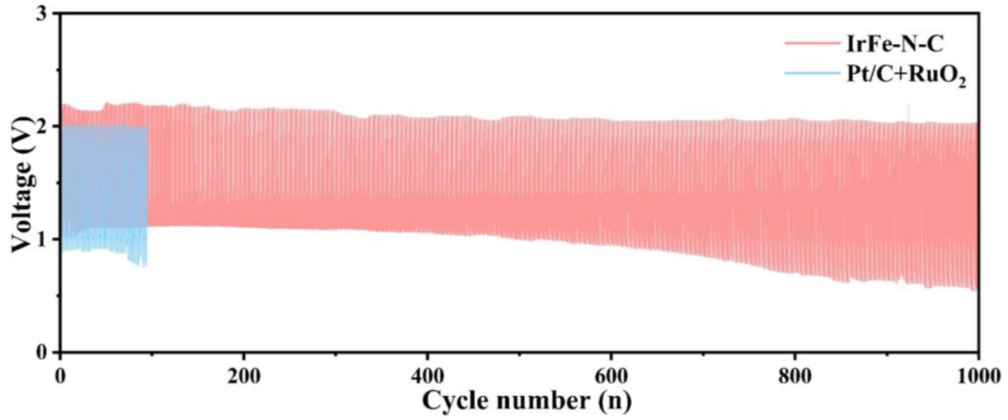

Figure S19. The cycling durabilty of the two-electrode rechargeable Zn–air battery with the IrFe–N–C air cathode under a current density of 10 mA cm^{-2}.

Table S1. The actual metal contents in Fe–N–C, Ir–N–C and IrFe–N–C.

Sample	Ir (wt.%)	Fe (wt.%)
**IrFe–N–C**	**1.62**	**1.29**
**Ir–N–C**	**1.04**	**—**
**Fe–N–C**	**—**	**0.97**

Table S2. Comparison of the OER performance for IrFe–N–C catalyst with other Ir-based electrocatalysts in alkaline solution.

Catalyst	Electrolyte	η_{10}[mV]	Reference	Ir loading [mg cm^{-2}]	Ref
IrFe–N–C	1 M KOH	227	This work	0.00842	—
Ir_{16}-PdCu/C	0.1 M KOH	284	ACS Nano 2021, 21, 13	0.00546	[1]
Ni-Ir0.5	1 M KOH	257	Inorg. Chem. Front. 2022, 9, 6225	1	[2]
Ir_3Ni_2/BMNC	1 M KOH	279	Adv. Mater. 2020, 7, 24	0.424	[3]
A-Ir_1/$Co_{0.8}Fe_{0.2}Se_2$	1 M KOH	230	Nat. Commun. 2020, 11, 1215	1	[4]
CoIr-0.2	1 M KOH	235	Adv. Mater. 2018, 30,18	0.566	[5]
NiFeIrO	1 M KOH	240	J. Electroanal. Chem. 2024, 967, 118424	0.0858	[6]
IrO_2/CNT	1 M KOH	249	ACS Catal. 2017, 7, 9	0.016	[7]
Surface-clean 3D Ir	1 M KOH	242	Nano Lett. 2016, 16, 7	0.0115	[8]
IrNi NCs	1 M KOH	270	Adv. Mater. 2017, 27, 27	0.0125	[9]
Ir-pZCO	1 M KOH	304	Inorg. Chem. Front. 2024, 11, 4876	0.7	[10]
IrW NDs	0.1 M KOH	300	ACS Cent. Sci. 2018, 4, 9	0.0102	[11]
CoIr/N-Ti	1 M KOH	260	ACS Appl. Nano Mater. 2025, 8, 10	0.13	[12]
RuIrOx	1 M KOH	250	Nat. Commun. 2019, 10, 4875	0.01	[13]
Ir@N-G	1 M KOH	270	Nano Energy. 2019, 62, 117	0.023	[14]
Ir-NSG	1 M KOH	307	Nat. Commun. 2020, 11, 4246	0.3	[15]

**Li-$IrSe_2$**	**1 M KOH**	**270**	**Angew. Chem Int. Ed. 2019, 58, 41**	**1**	**[16]**
**a-Ir/Vo-FeCo LDH**	**1 M KOH**	**203**	**Appl. Surf. Sci.2024, 665, 16031**	**1.33**	**[17]**
**Ir/$NiFe_2O_4$**	**1 M KOH**	**196**	**Small. 2025, 21, 24**	**0.087**	**[18]**
**Ir@Ni（OH）$_2$/FeOOH**	**1 M KOH**	**183**	**Appl. Surf. Sci. 2025, 700, 163249**	**1**	**[19]**
**Ir-CeO_2-C NFs**	**1 M KOH**	**279**	**Nano Res. 2023, 16, 5**	**1**	**[20]**
**Irlat@Co_3O_4**	**1 M KOH**	**285**	**J. Energy Chem. 2024, 89, 355–363**	**0.156**	**[21]**

Table S3. The comparison of OER performance and stability testing between IrFe–N–C catalysts and other Ir-based electrocatalysts in alkaline solutions.

**Catalyst**	**Electrolyte**	**η_{10}[mV]**	**Stability Test (@10 mA cm^{-2})**	**Reference**	**Ref**
**IrFe–N–C**	**1 M KOH**	**227**	**200 h**	**This work**	**—**
**Ir-NSG**	**1 M KOH**	**220**	**24 h**	**Nat. Commun. 2020, 11, 4246**	**[15]**
**$Ir_1Ni@MoO_2$**	**1 M KOH**	**280**	**100 h**	**Adv. Mater. 2024, 36, 2305437**	**[22]**
**Ir/Fe_2O_3@CNTs**	**1 M KOH**	**289**	**24 h**	**Nano Mater. Sci. 2025, 7, 100005**	**[23]**
**Ir@VOSe**	**1 M KOH**	**220**	**56 h**	**Adv. Funct. Mater. 2025, e12965**	**[24]**
**IrNi@NiFe-MOFs**	**1 M KOH**	**228**	**40 h**	**Adv. Funct. Mater. 2025, 35, 2500568**	**[25]**

Table S4. Comparison of bifunctional ORR/OER performance between IrFe–N–C catalyst and representative NiFe-based/Fe–N–C non-noble-metal benchmarks in alkaline electrolytes.

**Catalyst**	**Electrolyte**	**OER η_{10} [mV]**	**ORR $E_{1/2}$ [V]**	**ΔE（η_{10} - $E_{1/2}$）[V]**	**Reference**	**Ref**
**IrFe–N–C**	**1 M KOH**	**227**	**0.933**	**0.55**	**This work**	**—**
**NiFe-LDH/Fe_1-N-C**	**1 M KOH**	**320**	**0.9**	**0.65**	**Adv. Energy Mater. 2023, 2203609**	**[26]**
**Ni_2Fe_1@PANI-KOH900)**	**1 M KOH**	**240**	**0.92**	**0.55**	**J. Mater. Chem. A, 2019, 7, 19045**	**[27]**
**Fe_xNC@NiFe$(OH)_x$**	**1 M KOH**	**264**	**0.858**	**0.636**	**Inorg. Chem. Front., 2023, 10, 1758**	**[28]**
**Fe-NCNT@NiFe-LDH**	**1 M KOH**	**180**	**0.89**	**0.54**	**EES Catal., 2023, 1, 987**	**[29]**
**NiFe-LDH@NC**	**1 M KOH**	**310**	**0.83**	**0.72**	***Biochar*, 2023, 5, 60**	**[30]**
**FeNi LDH@DACs**	**0.1 M KOH**	**300**	**0.86**	**0.67**	**ACS Nano 2025, 19, 32231−32245**	**[31]**
**FeNi-NC@MWCNT**	**0.1 M KOH**	**370**	**0.9**	**0.69**	**Small 2025, 21, 2409161**	**[32]**
**Mo-NiFe LDH/Ti**	**1 M KOH**	**232**	**—**	**—**	**Adv. Sci. 2026, 13, e15407**	**[33]**
**V_{Cr}, Co-NiFeOOH**	**1 M KOH**	**198**	**—**	**—**	**J.Am.Chem.Soc.2025,147,2607−2615**	**[34]**

References

[1] Y. Qin, Z. Wang, W. Yu, Y. Sun, D. Wang, J. Lai, S. Guo, L. Wang, *Nano Lett.* **2021**, *21*, 5774.

[2] S. Yan, M. Zhong, W. Zhu, W. Li, X. Chen, M. Li, C. Wang, X. Lu, *Inorg. Chem. Front.* **2022**, *9*, 6225.

[3] X. Chen, M. Xu, S. Li, C. Li, X. Sun, S. Mu, J. Yu, *Adv. Mater. Interfaces* **2020**, *7*, 2001145.

[4] Z. Zhang, C. Feng, C. Liu, M. Zuo, L. Qin, X. Yan, Y. Xing, H. Li, R. Si, S. Zhou, J. Zeng, *Nat. Commun.* **2020**, *11*, 1215.

[5] Y. Zhang, C. Wu, H. Jiang, Y. Lin, H. Liu, Q. He, S. Chen, T. Duan, L. Song, *Adv. Mater.* **2018**, *30*, 1707522.

[6] Y. Zhu, C. Liu, H. Zhang, Z. Zhou, Y. Jiang, T. Wang, Y. Liu, *J. Electroanal. Chem.* **2024**, *967*, 118424.

[7] J. Guan, D. Li, R. Si, S. Miao, F. Zhang, C. Li, *ACS Catal.* **2017**, *7*, 5983.

[8] Y. Pi, N. Zhang, S. Guo, J. Guo, X. Huang, *Nano Lett.* **2016**, *16*, 4424.

[9] Y. Pi, Q. Shao, P. Wang, J. Guo, X. Huang, *Adv. Funct. Mater.* **2017**, *27*, 1700886.

[10] J. Chen, J. Liu, S. Xu, Y. Wu, Y. Ye, J. Qian, *Inorg. Chem. Front.* **2024**, *11*, 4876.

[11] F. Lv, J. Feng, K. Wang, Z. Dou, W. Zhang, J. Zhou, C. Yang, M. Luo, Y. Yang, Y. Li, P. Gao, S. Guo, *ACS Cent. Sci.* **2018**, *4*, 1244.

[12] A. Q. K. Nguyen, H. Q. Pham, Q. Huynh, T. T. Huynh, *ACS Appl. Nano Mater.* **2025**, *8*, 5121.

[13] Z. Zhuang, Y. Wang, C.-Q. Xu, S. Liu, C. Chen, Q. Peng, Z. Zhuang, H. Xiao, Y. Pan, S. Lu, R. Yu, W.-C. Cheong, X. Cao, K. Wu, K. Sun, Y. Wang, D. Wang, J. Li, Y. Li, *Nat. Commun.* **2019**, *10*, 4875.

[14] X. Wu, B. Feng, W. Li, Y. Niu, Y. Yu, S. Lu, C. Zhong, P. Liu, Z. Tian, L. Chen, W. Hu, C. M. Li, *Nano Energy* **2019**, *62*, 117.

[15] Q. Wang, C.-Q. Xu, W. Liu, S.-F. Hung, H. Bin Yang, J. Gao, W. Cai, H. M. Chen, J. Li, B. Liu, *Nat. Commun.* **2020**, *11*, 4246.

[16] T. Zheng, C. Shang, Z. He, X. Wang, C. Cao, H. Li, R. Si, B. Pan, S. Zhou, J. Zeng, *Angew. Chem. Int. Ed.* **2019**, *58*, 14764.

[17] X. Deng, J. Chen, Q. Chen, Y. Zhou, X. Liu, J. Zhang, G. Wang, R. Wang, *Appl. Surf. Sci.* **2024**, *665*, 160310.

[18] X. Duan, X. Yu, T. Yang, E. Wang, Y. Hou, X. Hou, *Small* **2025**, *21*, 2503136.

[19] M. Gan, Y. Song, J. Wei, Y. Shen, P. Liu, M. Xia, P. Zhang, Z. Tian, B. Xu, J. Guo, *Appl. Surf. Sci.* **2025**, *700*, 163249.

[20] X. Chen, W. Liao, M. Zhong, J. Chen, S. Yan, W. Li, C. Wang, W. Chen, X. Lu, *Nano Res.* **2023**, *16*, 7724.

[21] W. Chen, Y. Song, L. Li, J. Guo, Z. Lin, *J. Energy Chem.* **2024**, *89*, 355.

[22] B. Wang, J. Li, D. Li, J. Xu, S. Liu, Q. Jiang, Y. Zhang, Z. Duan, F. Zhang, *Adv. Mater.* **2024**, *36*, 2305437.

[23] X. Sun, D. Zhang, K. Xu, B. Qian, K. Chen, D. Xue, *Nano Materials Science* **2025**, 7, 100005.

[24] J. Mei, J. Zhao, M. Li, J. Shang, Q. Yao, J. Bai, S. Dou, *Adv. Funct. Mater.* 2025, e12965.

[25] J. Jia, Y. Wang, Y. Cha, Z. Wang, J. Huang, D. Wang, H. Li, K. Guo, J. Li, J. Huang, Y. Tang, C. Xu, *Adv. Funct. Mater.* **2025**, *35*, 2500568.

[26] Z. Q. Liu, X. Liang, F. X. Ma, Y. X. Xiong, G. Zhang, G. Chen, L. Zhen, C. Y. Xu, *Adv. Energy Mater.* **2023**, *13* , 2203609.

[27] J. Zhang, M. Zhang, L. Qiu, Y. Zeng, J. Chen, C. Zhu, Y. Yu, Z. Zhu, *J. Mater. Chem. A* **2019**, *7* , 19045.

[28] H. Luo, Y. Li, W. Wang, T. Zhou, Z. Guo, *Inorg. Chem. Front.* **2023**, *10* , 1758.

[29] J. Li, N. Huang, M. Lv, N. Su, C. Li, Y. Huang, Y. Wang, Y. Zheng, W. Liu, T. Ma, L. Ye, *EES Catal.* **2023**, *1* , 987.

[30] P. Zhang, K. Sun, Y. Liu, B. Zhou, S. Li, J. Zhou, A. Wang, L. Xie, B. Li, J. Jiang, *Biochar* **2023**, *5* ,60.

[31] T. Qin, L. Zheng, Z. Pei, W. Wang, X. Ouyang, Z. Xu, J. Wang, X. Guo, J. Lu, *ACS Nano* **2025**, *19* , 32231.

[32] Z. Chen, W. Cheng, K. Cao, M. Jin, S. Rahali, S. A. Chala, E. Ebrahimi, N. Ma, R. Liu, K. Lakshmanan, C. Y. Chang, C. C. Cheung, H. Luo, Y. Wang, B. J. Hwang, C. Streb, *Small* **2024**, *21* , 2409161.

[33] S. Wu, W. Lu, S. Zhao, K. Zhao, N. Yan, L. Huang, D. Li, T. Jiang, H. Wu, F. Ren, *Advanced Science* **2025**, *13* , e15407.

[34] H.-J. Niu, N. Ran, W. Zhou, W. An, C. Huang, W. Chen, M. Zhou, W.-F. Lin, J. Liu, L. Guo, *J. Am. Chem. Soc.* **2025**, *147* , 2607.
